# Pilot Quality-Assurance Study of a Third-Generation Batch-Mode Clinical-Scale Automated Xenon-129 Hyperpolarizer

**DOI:** 10.3390/molecules27041327

**Published:** 2022-02-16

**Authors:** Jonathan R. Birchall, Md Raduanul H. Chowdhury, Panayiotis Nikolaou, Yuri A. Chekmenev, Anton Shcherbakov, Michael J. Barlow, Boyd M. Goodson, Eduard Y. Chekmenev

**Affiliations:** 1 Department of Chemistry, Integrative Biosciences (Ibio), Karmanos Cancer Institute (KCI), Wayne State University, Detroit, MI 48202, USA; raduanul@wayne.edu; 2 XeUS Technologies Ltd., Nicosia 2312, Cyprus; peternikolaou78@gmail.com (P.N.); yura_chekmenev@mail.ru (Y.A.C.); 3 Smart-A, 614000 Perm, Russia; xbister@gmail.com; 4 Custom Medical Systems (CMS) Ltd., Nicosia 2312, Cyprus; 5 Sir Peter Mansfield Imaging Centre, University of Nottingham, Nottingham NG7 2RD, UK; michaelj.barlow@me.com; 6 Department of Chemistry and Biochemistry, Materials Technology Center, Southern Illinois University, Carbondale, IL 62901, USA; bgoodson@chem.siu.edu; 7 Russian Academy of Sciences, Leninskiy Prospekt 14, 119991 Moscow, Russia

**Keywords:** NMR, hyperpolarization, MRI, Xenon-129, Xenon, spin exchange optical pumping, hyperpolarizer, automation, quality assurance, polarimetry

## Abstract

We present a pilot quality assurance (QA) study of a clinical-scale, automated, third-generation (GEN-3) ^129^Xe hyperpolarizer employing batch-mode spin-exchange optical pumping (SEOP) with high-Xe densities (50% natural abundance Xe and 50% N_2_ in ~2.6 atm total pressure sourced from Nova Gas Technologies) and rapid temperature ramping enabled by an aluminum heating jacket surrounding the 0.5 L SEOP cell. ^129^Xe hyperpolarization was performed over the course of 700 gas loading cycles of the SEOP cell, simulating long-term hyperpolarized contrast agent production in a clinical lung imaging setting. High levels of ^129^Xe polarization (avg. %*P*_Xe_ = 51.0% with standard deviation σ*_P_*_Xe_ = 3.0%) were recorded with fast ^129^Xe polarization build-up time constants (avg. *T*_b_ = 25.1 min with standard deviation σ*_T_*_b_ = 3.1 min) across the first 500 SEOP cell refills, using moderate temperatures of 75 °C. These results demonstrate a more than 2-fold increase in build-up rate relative to previously demonstrated results in a comparable QA study on a second-generation (GEN-2) ^129^Xe hyperpolarizer device, with only a minor reduction in maximum achievable %*P*_Xe_ and with greater consistency over a larger number of SEOP cell refill processes at a similar polarization lifetime duration (avg. *T*_1_ = 82.4 min, standard deviation σ*_T_*_1_ = 10.8 min). Additionally, the effects of varying SEOP jacket temperatures, distribution of Rb metal, and preparation and operation of the fluid path are quantified in the context of device installation, performance optimization and maintenance to consistently produce high ^129^Xe polarization values, build-up rates (*T*_b_ as low as 6 min) and lifetimes over the course of a typical high-throughput ^129^Xe polarization SEOP cell life cycle. The results presented further demonstrate the significant potential for hyperpolarized ^129^Xe contrast agent in imaging and bio-sensing applications on a clinical scale.

## 1. Introduction

Nuclear magnetic resonance (NMR) has seen widespread application in the fields of anatomical and physiological clinical imaging. Magnetic resonance imaging (MRI) techniques are advantageous by virtue of their non-invasive, non-ionizing nature, as well as their good spatial and temporal resolution and superb contrast in soft tissues. One area where traditional MRI techniques are less effective, however, is in the study of lung structure and function. This is primarily due to the inherently low density of requisite proton nuclear spins and *B*_0_ inhomogeneities in lung air sacks, coupled with the low degree of nuclear spin alignment with the applied static magnetic field, i.e., nuclear spin polarization (*P*). For example, in vivo proton *P* (*P*_H_) at thermal equilibrium is only ~1 × 10^−5^ in modern 3 T MRI scanners.

Hyperpolarization of NMR-active nuclear species such as ^3^He, ^129^Xe, and other gases can overcome both of these issues. Hyperpolarization techniques are capable of boosting *P* to the order of unity, resulting in five or more orders of magnitude increase in the detection sensitivity of MR spectroscopic and imaging techniques [1,2,3,4]. Such gains in sensitivity facilitate detection of these hyperpolarized (HP) nuclear species in the form of inhalable MR contrast agents [5,6,7,8,9,10,11,12,13,14,15,16], as well as other applications related to molecular sensing [8,9,17,18,19,20]. Their relative safety and suitability for this task are underpinned by both the inert nature and the low natural abundance of ^3^He and ^129^Xe, minimizing background signal from non-HP nuclei.

Since straightforward brute force hyperpolarization techniques, reliant on strong magnetic fields (>10 T) and low temperatures (T → 0 K), are relatively impractical in an in vivo clinical imaging setting, alternative approaches have been developed to achieve sufficient MR signal enhancement. In the case of HP ^129^Xe contrast agent production, the spin-exchange optical pumping (SEOP) process is typically employed. In this two-stage process, circular polarization is transferred from laser photons to ^129^Xe nuclei via Fermi contact interactions with an intermediary electronic alkali metal (e.g., rubidium) vapor [21,22]. There are two available modalities for HP ^129^Xe contrast agent production, each with their own advantages and drawbacks [23,24]. The first of these is the “continuous-flow” modality [25,26,27,28,29], whereby a continuous flow of a ^129^Xe-containing gas mixture at low Xe densities is hyperpolarized to high ^129^Xe *P* (*P*_Xe_), with the HP ^129^Xe exiting the sealed vessel (termed “SEOP cell”) and being collected on a cryogenically-cooled cold finger. The frozen HP ^129^Xe is then thawed and dispensed in a Tedlar bag, where it can be transiently stored in the gas phase for up to a few hours prior to administration to a human subject. The second modality is a “batch-mode” (stopped-flow) SEOP approach [30,31,32,33,34,35,36,37], whereby a fixed volume of ^129^Xe-containing gas mixture at high Xe densities is hyperpolarized to high *P*_Xe_ inside the SEOP cell, which is typically pressurized over 1 atm. After *P*_Xe_ is built up over time, the HP ^129^Xe gas mixture can then be ejected into a Tedlar bag without the need for cryo-collection [34,35,38,39].

In addition to high levels of *P*_Xe_, either approach can be utilized to produce HP ^129^Xe gas mixtures in clinically relevant volumes (~1 L or more) and on timescales (<60 min) for imaging studies [23,25,28,35,39,40,41,42,43]. Inhalation of HP ^129^Xe in this way enables the determination of lung morphology and function via MRI in both human and animal subjects for a wide variety of purposes [7,43,44,45,46,47]. In addition to lung imaging, the solubility of ^129^Xe in blood allows for this source of hyperpolarization to traverse the alveolar wall and even the blood–brain barrier, additionally facilitating imaging of the brain [48,49,50,51], brown fat [52,53], and other bio-sensing [17,19,54,55]. The electronic structure of ^129^Xe bolsters this advantage by demonstrating measurable changes in chemical shift in response to even small changes in the local environment, providing a novel sensing mechanism.

Despite the inert nature of the typical Xe/N_2_ gas mixture utilized in the production of HP ^129^Xe contrast agents, the procedure is not without some risk. In addition to possessing anesthetic properties, Xe is as a dense gas, and inhalation of sufficiently large quantities has the potential for asphyxiation as oxygen necessary for respiration is displaced. Therefore, in all of the above-mentioned applications in the field of biomedical imaging, HP ^129^Xe is considered a drug, and as such, production and use are highly regulated and controlled. Producing large quantities of highly-polarized contrast agent in a short time-span is obviously important, but it is also important to ensure a high degree of consistency between subsequent production cycles to demonstrate excellent reproducibility, and along with it, build trust in the technology.

Although modern ^129^Xe hyperpolarizer devices utilize ultra-high-purity (UHP) gas mixture sources and inert gas purifiers, these cannot eliminate the presence of impurities completely. Oxygen, water vapor, and other gases may remain in trace quantities, or may ingress from minute leaks from the atmosphere. Over the course of many production cycles, these contaminants will bind with the highly-reactive spin-exchanging alkali metal intermediary, causing the system to degrade in performance. This degradation happens firstly because the presence of small quantities of alkali metal oxide and hydroxide can increase the Rb boiling point compared to that of the pure metal; this change in the colligative properties reduces the alkali metal vapor pressure, and by extension, the efficiency of pump laser absorption. Additionally, the presence of paramagnetic centers results in more rapid relaxation of passing ^129^Xe nuclei, decreasing the effective longitudinal spin relaxation time constant (*T*_1_) within the SEOP cell. Both of these possibilities result in a detrimental effect on the maximum achievable net spin polarization (%*P*_Xe_) [56,57,58].

Regeneration of SEOP cells containing slightly Rb is possible to a certain extent with high-heat temperature-cycling methods [40], but complete recovery is difficult to achieve. Eventually, SEOP cells will need to be replaced by new ones containing fresh Rb (note the process of Rb refilling of the SEOP cell is a time- and labor-intensive operation spanning several days to ensure sufficient SEOP cell purity). This potential for disruption to operation is particularly problematic when applied to clinical-scale imaging operations, where subjects may need to undergo imaging at scheduled intervals to monitor disease progression or treatment efficacy.

Following on from our previous second-generation (GEN-2) stopped-flow ^129^Xe hyperpolarizer [59,60,61] quality-assurance (QA) study [40], we present an expanded pilot QA assessment of a third-generation (GEN-3) automated batch-mode ^129^Xe hyperpolarizer [39,62] under clinically-relevant conditions of operation. The central motivation for this work is two-fold. First, we determine the effective range of the device parameters in the context of robust operation. Second, we demonstrate the feasibility of refilling the SEOP cell gas mixture many hundreds of times in order to simulate clinical-scale production of HP ^129^Xe. More specifically, we investigated a course of 700 SEOP cell refills—Xe/N_2_ interleaved with UHP N_2_ to reduce waste and make experimentation time more feasible—with all experiments described being conducted within a timespan of two months: Over the course of the first 500 refills, we observed negligible decrease in maximum achievable ^129^Xe polarization (%*P*_Xe_), as well as negligible changes in either polarization build-up rates or relaxation time constants (*T*_1_). All SEOP cell refills were complete, i.e., the cell content was evacuated to 5 × 10^−2^ Torr prior to receiving a fresh load of gas. Hyperpolarization efficiency and effective HP ^129^Xe *T*_1_ in the SEOP cell were observed to reduce over the course of the final 200 SEOP cell refills, but not to terminal levels where insufficient levels of useful ^129^Xe polarization were recorded. This study is supplemented by a variety of miscellaneous QA-related measurements, including time- and temperature-dependent mapping of NMR radio frequency (RF) excitation pulse duration (employed for in situ *P*_Xe_ monitoring), off-resonance RF excitation, and magnetic field calibration. In addition to comparing ^129^Xe hyperpolarization build-up efficiency as a function of SEOP cell temperature, we also describe how variation in other polarizer-specific parameters, such as the time interval between NMR acquisitions (i.e., repetition time of NMR data sampling) and the amount of cooling power supplied to external fans on the device impact operation of the hyperpolarizer performance.

This work demonstrates that the high reproducibility demanded for clinical-scale HP ^129^Xe contrast agent production is achievable on our automated GEN-3 hyperpolarizer device, as well as provides a guide concerning good practice for mapping the operational conditions of a ^129^Xe hyperpolarizer to enable a device’s robust and efficient installation and operation in both clinical and research environments. These procedures are intended to be applicable to many designs of batch-mode hyperpolarizers [34,35,37,38,39] regardless of site or scale, and should be of interest to those seeking to determine the feasibility of efficiently scaling up ^129^Xe hyperpolarization from a laboratory setting to a clinical one. Future potential improvements of the GEN-3 hyperpolarizer are also discussed.

## 2. Materials and Methods

### 2.1. GEN-3 ^129^Xe Hyperpolarizer Overall Design

**Figure 1:**
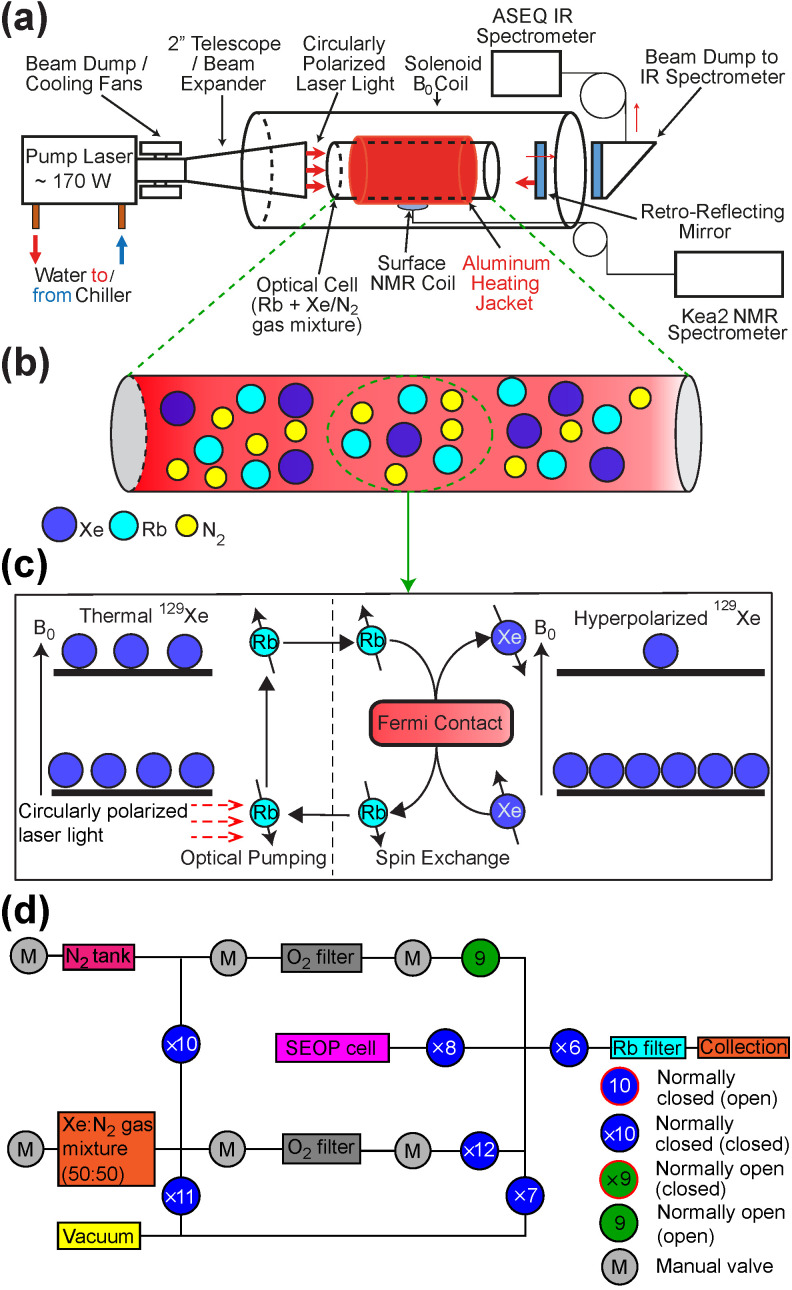
(**a**) Schematic of the GEN-3 ^129^Xe hyperpolarizer; (**b**) gaseous species involved in the SEOP process; (**c**) ^129^Xe SEOP process diagram; (**d**) GEN-3 ^129^Xe hyperpolarizer gas-handling manifold providing the connection of the valves and their numbers. “Normally open (open)” refers to the valve being open in the de-energized state; “normally closed (closed)” refers to the valve being closed in the de-energized state; “normally closed (open)” refers to the valve being open in the energized state; “normally open (closed)” refers to the valve being closed in the energized state. The numbers inside the circles indicate the numbering of the valves, which is employed for automated QA sequence actuation (see the text and figures below for details).

The GEN-3 ^129^Xe hyperpolarizer is an automated, batch-mode configuration device that can perform rapid and robust HP ^129^Xe gas production via the SEOP process. Figure 1a schematically displays the GEN-3 hyperpolarizer upper chassis, which illustrates the essential components required for performing the SEOP process and also to perform acquisition of in situ NMR and IR data, respectively required for the calculation of nuclear ^129^Xe spin polarization (%*P*_Xe_) and electronic Rb polarization (%*P*_Rb_). Figure 1b,c depict the SEOP processes that takes place inside the sealed SEOP cell. The automated nature of the GEN-3 hyperpolarizer enables the control of all essential components, e.g., optical pumping laser, SEOP cell jacket temperature, *B*_0_ solenoid coil current, and gas-handling manifold solenoid valves. All of these parameters can be configured from an easily-accessible graphical user interface (GUI), operating on a custom-built micro-controller driver module. A GUI screenshot is provided in Figure S8 in the Supporting Information (SI).

**Figure 2:**
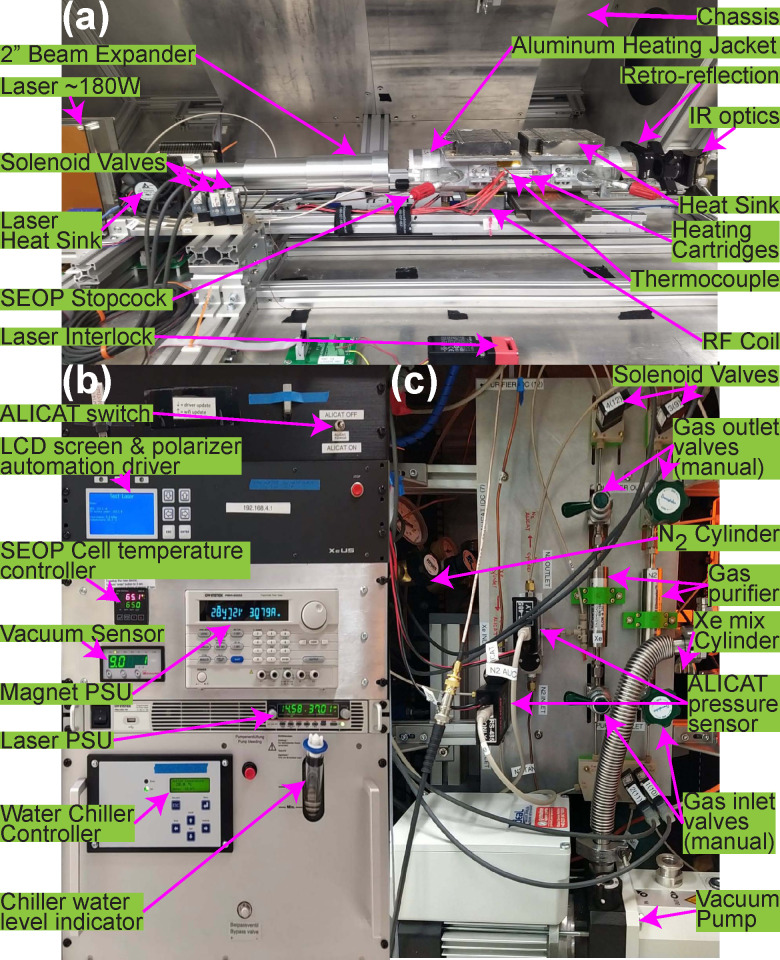
Annotated photographs of the GEN-3 ^129^Xe hyperpolarizer: (**a**) upper chassis with magnetic solenoid coil removed to show the SEOP cell, NMR coil, and heating jacket, (**b**) front panel housing the driver module, pump laser and magnetic solenoid coil power supply units, temperature and vacuum sensors, and water chiller, and (**c**) side panel housing the gas-handling manifold, gas cylinders and vacuum pump.

An annotated photo of the device is shown in Figure 2. The Rb-loaded SEOP cell (discussed in more detail in Section 2.2) is placed inside the aluminum jacket/cell holder of the GEN-3 hyperpolarizer [39] with an external coating of Arctic Alumina thermal paste (thermal conductivity ~4 W·K^−1^·m^−1^ at 25 °C and suitable for operation from −40 to 160 °C), ensuring good thermal contact between the SEOP cell and the aluminum jacket (see Figure 2a). The combination of the aluminum jacket and thermal paste distributes heat application from the heating element to the jacket extremities. Four 6 mm diameter heating cartridges (50 W each) are attached to the jacket via aluminum couplers. The cartridges are used for heating the SEOP cell—temperature ramping from ~25 to ~75 °C can be accomplished within 4 min [39]. The high thermal conductivity of aluminum allows dissipating the heat uniformly along the 7 mm thick jacket body. Heating cartridge operation is controlled by a PID controller (P/N 16B-23, Dwyer Instruments).

Cooling of the SEOP cell is achieved by using three heat sinks adjacent to the aluminum jacket. The circulating air removes excess of heat. The cooling process is facilitated by the dissipation of excess heat outside of the polarizer with the help of ten independent fans attached to the upper chassis of the polarizer. The degree of fan operation can be varied during the SEOP hyperpolarization process and during SEOP cell cooldown (see Figure S6); keeping all fans on facilitates a faster cooling process, whereas keeping fewer fans on during SEOP allows for less cartridge heating power to be utilized. The air ventilation design employed here helps to mitigate Rb runaway [63,64,65,66], an undesirable phenomenon whereby the cell temperature and Rb density rapidly increase in a self-reinforcing fashion, destabilizing SEOP and depolarizing the Xe (because the Rb-dense cell becomes opaque to the laser, impeding Rb polarization). Temperature measurement of the SEOP cell surface is performed by using a non-magnetic TKX-type thermocouple (FTC Foil Thermocouple, FluxTeq, Blacksburg, VA, USA).

Circularly-polarized photons (>98% polarization) are supplied by a continuous-wave (CW) pump laser (nominal ~170 W (actual ~140–150 W), Bright-Lock Ultra-500, QPC Laser Technologies, Sylmar, CA, USA), for which maximum driving current for long-term use is 37.5 A. A beam expanding telescope and collimating lens of 2” is used to match the laser output to the diameter of the SEOP cell to help ensure uniform SEOP cell illumination (telescope also provides circular polarization via a ¼ wave plate). The pump laser full width at half maximum (FWHM) was 0.154 nm. The laser resonance was tuned to the Rb D_1_ absorption wavelength of 794.77 nm with the help of a thermistor and P312 water chiller (Termotek, Baden-Baden, Germany). The water chiller used for this device runs with a flow rate of >4 L/min and provides 930 W of cooling power. Along with the laser heat sink, the water chiller plays an integral role in maintaining a stable and safe laser temperature, as overheating can cause irreparable damage to the laser diode array (LDA).

A *B*_0_ magnetic solenoid coil (~300 ppm field variance over a 5 cm sphere with up to 3.6 mT magnetic field) surrounds the aluminum jacket and the SEOP cell to produce a field that enables efficient SEOP, while providing sufficient homogeneity for sensitive NMR. The NMR data were collected using a RF surface coil positioned underneath the SEOP cell using a low-frequency Kea2 NMR spectrometer (Magritek, Wellington, New Zealand). An RF pulse amplifier (250 W, BT00250-AlphaA, Tomco Technologies, Stepney, Australia) was used to deliver the desired *B*_1_ RF pulse strength. The RF coil employs a parallel LC circuit optimized for detection at 40.8 kHz, with a resistance R = 20 Ohm (X_R_ = 20 Ohm), a tuning capacitor of C_T_ = 33,000 pF with impedance XC of ~130 Ohm at 40 kHz, and a multi-turn inductor of L = 0.5 mH with impedance X_L_ of ~130 Ohm at 40 kHz (total impedance of 280 Ohm). The RF coil is encased in an aluminum enclosure that is directly mated to the aluminum jacket; the RF coil faces the SEOP cell through a rectangular opening in the jacket. This shielded design eliminates much of the noise from the surroundings, which alternatively can be suppressed using a noise-canceling RF coil operating at the same frequency [38].

Near-infrared (NIR) spectroscopy was performed to estimate the rubidium polarization, %*P*_Rb_, as well as monitor the pump laser absorption status in real-time with a high-resolution optical spectrometer (HR1-B, ASEQ Instruments, Vancouver, Canada). At the rear of the SEOP cell, a mirror is used to retro-reflect the laser beam to provide a second pass through the SEOP cell. NMR and NIR data were processed automatically using a MATLAB software package described previously [67]. This automated open-source software package is freely available in the electronic supplemental information of Ref. # [67].

Vacuum and gas handling is performed using a custom manifold equipped with solenoid valves (type 6126, Burkert, Germany), Figure 1d and Figure 2c. These valves are mounted on four polyether ether ketone (PEEK) bases. Manual valves are additionally employed to gate the gas purifiers, reducing the minute amount of contamination from gas mixtures during filling. We anticipate eliminating the manual gating valves for the future operation. All gas/vacuum handling, as well laser power, water chiller, magnet PSU, fans, and SEOP cell temperature are controlled via a Wi-Fi enabled graphical user interface (GUI).

### 2.2. SEOP Cell Design

The core of the GEN-3 hyperpolarizer device is the SEOP cell that is used to produce HP ^129^Xe gas. The SEOP cell (made from Pyrex glass) has an internal volume of 500 mL and the diameter of 2″ (Mid Rivers Glassblowing, Saint Peters, MO, USA), which matches the expanded pump laser beam. Preparation and Rb filling protocols of the SEOP cell can be found elsewhere [39]. Briefly, the cell glass surface is extensively cleaned with a base solution (potassium hydroxide in methanol) and treated with a solution of Surfasil™ siliconizing agent in hexane to create a protective surface layer on the inner cell wall. This serves to reduce the effects of depolarizing collisions between HP ^129^Xe nuclei and paramagnetic centers within the walls of the SEOP cell, enhancing ^129^Xe in-cell *T*_1_—often to >2 h [39]. After the preparation and filling of the SEOP cell with ~0.5 g of Rb, the Rb metal was distributed across the inner surface by applying heat (using a heat gun) on one side while applying cooling on the exterior SEOP cell surface (e.g., by using dry ice) to condense the Rb vapor. Distributing the Rb metal as a high-surface-area film along the cell surface facilitates the process of creating a high-density vapor when the cell is heated for SEOP, as discussed below (see also Figures S4 and S5 in the SI; Figures S4 and S11 provide an annotated photo and the gas manifold schematic of the Rb “spreading”/distribution setup, respectively). The SEOP cell is closed from the external environment with the help of two independent stopcocks. We only use one of the stopcocks for gas and vacuum manipulations when the SEOP cell is installed within the hyperpolarizer. The second stopcock is helpful during SEOP cell cleaning and preparation. After placing the SEOP cell inside the aluminum heating jacket, PEEK tubing (1/8 in. OD) is employed to connect one side of the SEOP cell to the inlet valve of the gas manifold—see valve 8 in Figure 1d.

The SEOP process was performed with the stopcock in either an open or closed configuration. For the closed configuration, the stopcock is closed after filling the SEOP cell with a Xe/N_2_ gas mixture, and then the SEOP process is performed. The ‘stopcock-open’ configuration is useful for production and ejection of HP ^129^Xe and SEOP cell reloading with fresh, unpolarized gas, because the cell must be mated to the rest of the gas-handling manifold for these operations. The ‘stopcock-closed’ configuration seals the SEOP cell entirely from the outside environment for the long-term (weeks) storage to minimize the ingress of minute amount of impurities into the SEOP cell.

### 2.3. GEN-3 ^129^Xe Hyperpolarizer Calibration

**Figure 3:**
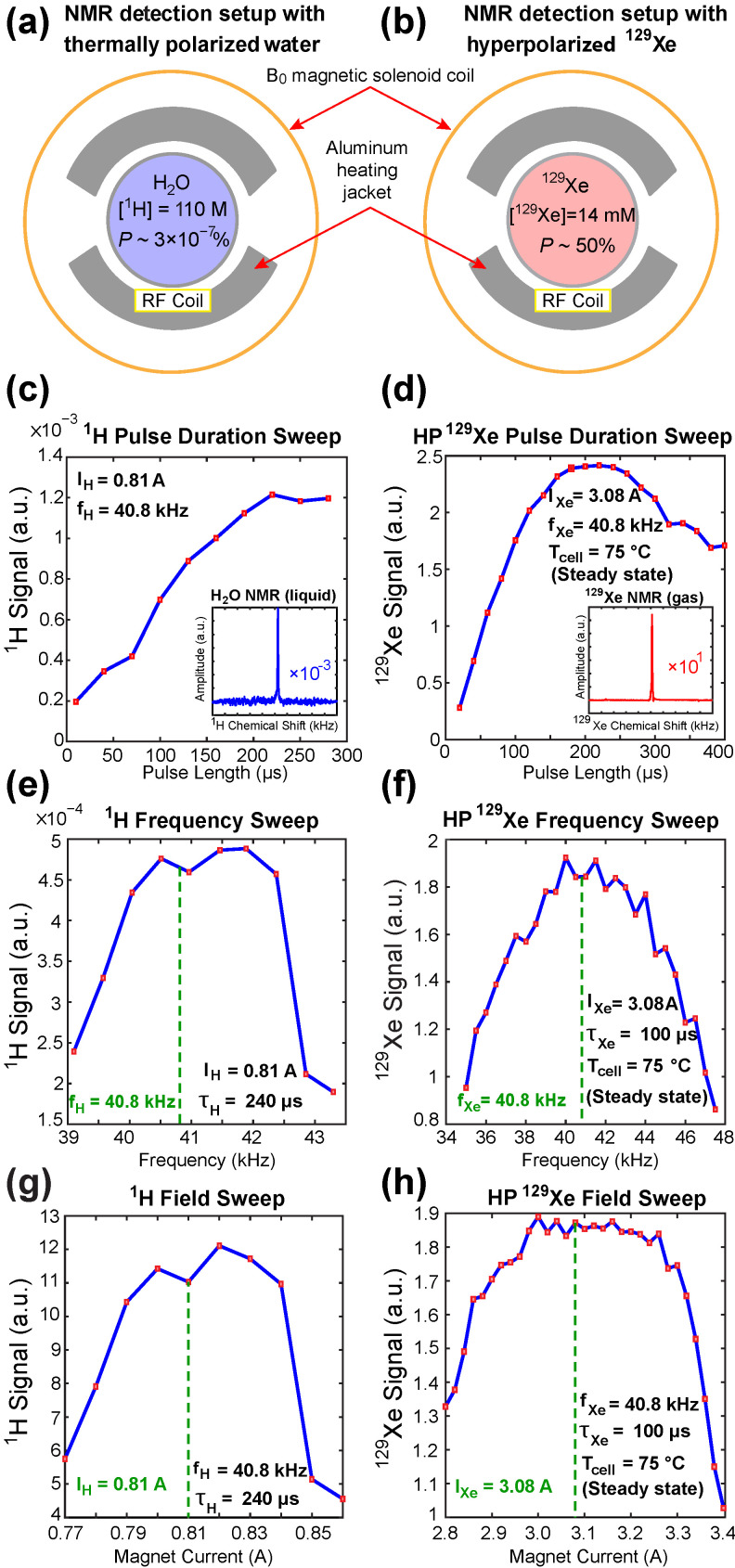
Schematic representation of the ^1^H (**a**) and ^129^Xe (**b**) sample arrangements. Displays (**c**,**d**) show RF pulse duration sweep for thermal ^1^H and HP ^129^Xe, respectively, with NMR spectra shown as inserts. Displays (**e**,**f**) show NMR signal intensity as a function of resonant frequency for thermal ^1^H and HP ^129^Xe, respectively, at fixed magnetic field current. Displays (**g**,**h**) show NMR signal intensity as a function of magnetic field driver current for thermal ^1^H and HP ^129^Xe samples, respectively. For protons, the ~1 mT field is swept over the 10% range reflecting the maximum possible fluctuation due to polarizer magnet alignment with the Earth’s magnetic field of ±0.05 mT. Note, RF amplifier outputs a 3.6-fold higher voltage for ^129^Xe RF excitation pulses compared to that for proton RF pulses to compensate for the difference in gyromagnetic ratios of the nuclei.

For computation of %*P*_Xe_, we compare the signal from HP ^129^Xe to that of a signal reference phantom with the same geometry and known concentration and nuclear spin polarization (see Section 2.4). In our device, we perform NMR detection of HP ^129^Xe and the thermally polarized proton signal reference phantom (500 mL water doped (placed in the same shaped glassware) with 10 mM CuSO_4_; ^1^H *T*_1_ ~ 0.05 s) at the same resonance frequency, 40.8 kHz (Figure 3a,b)—achieved by adjusting the electromagnetic field current to compensate for the ~3.6-fold difference between the ^1^H and ^129^Xe gyromagnetic ratios (γ_1H_ and γ_129Xe_). While in principle, the magnetic field can be adjusted with the aid of a gaussmeter, in practice, the precision of typical gaussmeters is generally insufficient for this task. This practical challenge was also confronted in the context of low-field parahydrogen induced hyperpolarizers [68], because the *B*_1_ strength of RF pulses and the quality factor of the RF coil itself allows robust operation only in a limited resonance frequency range. Therefore, two experimental challenges must be addressed in the context of quantitative comparison of the HP signal and thermally polarized reference signal with the goal to robustly determine %*P*_Xe_. The first one of these challenges is precise calibration of the electromagnet current to ensure on-resonance condition, and the second one is identification of a frequency and electromagnet current range with robust device operation. The latter is important in the context of operation at off-resonance conditions due to ambient field variance.

The HP ^129^Xe signal can be readily detected with a single shot, due to the significant signal enhancement (7–8 orders of magnitude) arising from hyperpolarization. Calibration of the RF pulse duration (τ_Xe_), *B*_0_ electromagnet current (I_Xe_), and resonant frequency (f_Xe_) was performed using single-shot NMR spectroscopy of steady-state HP ^129^Xe after %*P*_Xe_ build-up in a 1000 Torr Xe/1000 Torr N_2_ SEOP cell (Figure 3). Note, when the thermally polarized water phantom was employed, NMR signal was decreased by ~4 orders of magnitude, thus requiring signal averaging. In total, ~20,000 scans were employed to obtain a good signal-to-noise ratio (SNR)—see Figure 3c (inset). This approach facilitated calibration of RF pulse duration (τ_H_), *B*_0_ electromagnet current (I_H_), and resonant frequency (f_H_). CuSO_4_ doping enabled the repetition time to be reduced to 0.3 s. The ratio of I_Xe_ and I_H_ corresponding to the precise resonance conditions of the two spins may deviate from γ_1H_/γ_129Xe_ due to the presence of the Earth’s magnetic field; therefore, a magnetic field sweep should be performed for both nuclei during the device installation process. On-resonance condition for ^129^Xe (f_Xe_ = 40.8 kHz at ~3.6 mT) was found with I_Xe_ = 3.08 A.

Application of RF pulses with larger effective pulse tipping angle leads to increased depolarization of HP ^129^Xe nuclei within the SEOP cell, and frequent application may alter apparent parameters measured during SEOP process (e.g., reduced apparent *T*_1_ relaxation time constants—see below). Therefore, it is important to determine an appropriate balance between the amount of NMR signal generated by each pulse and polarization consumption. Because γ_1H_ ≈ 3.6⋅γ_129Xe_, the RF power of the ^129^Xe excitation RF pulse was increased by (3.6)^2^ in order to achieve the same B_1_ strength (and by extension spatial RF excitation profile) for both spins. Note, when the RF power (measured as (V_RMS_)^2^/50, where V_RMS_ = Vpp/2/√2; Vpp is the voltage of the oscilloscope set to 50 Ohm load) is mis-calibrated, the non-identical *B*_1_ fields applied to ^1^H and ^129^Xe spins lead to an over- or under-estimate of measured *P*_Xe_; for example, Figure S9 shows an example of using “stronger than required” ^129^Xe *B*_1_, leading to overestimation of *P*_Xe_ by a factor of 1.22. Based on the RF pulse calibrations shown in Figure 3c,d, we selected τ_H_ = τ_Xe_ = 150 μs as the pulse length for the subsequent QA studies. In some cases, where signal quantification for polarimetry purposes is not required (e.g., Figure 3), other pulse durations were employed that we tailored to the need of the experiment. For example, the water experiments used a pulse length of τ_H_ = 240 μs to maximize the otherwise-limited SNR.

Furthermore, we also performed resonance frequency sweeps (Figure 3e,f) and electromagnet current sweeps (Figure 3g,h). Besides measuring the respective Larmor frequencies and resonance electromagnet current values for ^1^H and ^129^Xe, these plots also provide insights about the robust ranges of operation for the electromagnetic solenoid coil. In the context of resonance frequency, the linear range of operation is ~2 kHz in both cases, likely reflecting limitations imposed by the RF coil quality factor, Q (estimated at ~30). With respect to the electromagnet current, the linear range of operation is ~0.05 A for ^1^H and 0.25 A for ^129^Xe. These operational ranges ensure the ability to perform robust and reproducible measurements of corresponding NMR signals. For this study, we used 0.810 A and 3.080 A current values for ^1^H and ^129^Xe spins, respectively, to reach the nominal 40.8 kHz resonance frequency for both spins.

### 2.4. SEOP Process

The photons supplied by the pump laser are absorbed by the outer-shell electron of Rb vapor atoms inside the SEOP cell. This absorption happens at the Rb D_1_ wavelength (794.77 nm, air referenced). Rb evaporation is much more efficient once the temperature is above the melting point (~39.3 °C [69]), allowing one to establish a sufficiently high concentration in the gas phase to induce laser light absorption. Nevertheless, the absorption of laser photons may still be limited at lower SEOP cell temperatures, as the Rb inside the SEOP cell may have limited surface distribution. As a result, the process to establish equilibrium gas-phase concentration (at a given temperature) may be kinetically limited due to small surface area of the Rb metal layer on SEOP cell surfaces. To mitigate this practical experimental limitation, it is often helpful to distribute the Rb metal as a thin film to increase the effective surface area inside the SEOP cell (see SI for more details).

**Figure 4:**
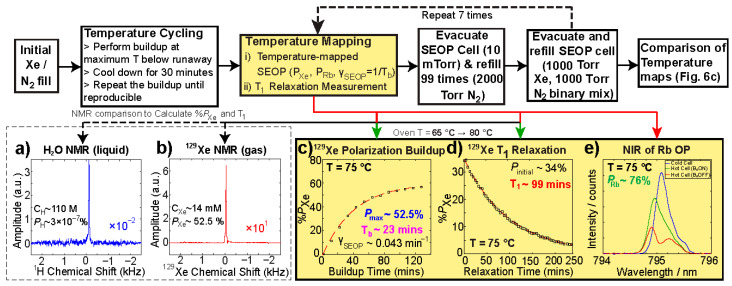
Synopsis of SEOP cell reloading quality assurance (QA) study consisting of 700 total SEOP cell gas mixture refills. ^129^Xe polarization (%*P*_Xe_) is calculated by comparing the peak area integral of HP ^129^Xe NMR spectra (**b**) with thermally-polarized ^1^H reference spectra (**a**). This process is repeated at fixed intervals to observe ^129^Xe polarization build-up (**c**) and decay (**d**) over time, respectively, providing a build-up rate (γ_SEOP_) and a relaxation time constant (*T*_1_) for each corresponding curve. Comparison of NIR pump laser spectra (**e**) facilitates calculation of an estimate of the average Rb polarization (%*P*_Rb_) throughout the cell. Key performance indicator (KPI) variables include steady-state %*P*_Xe_, γ_SEOP_, *T*_1_, and %*P*_Rb_, which can also be plotted as a function of SEOP cell temperature, as described in the text.

Once the SEOP cell preparation has been completed and the cell loaded with a gas mixture (nominally 1000 Torr Xe/1000 Torr N_2_; the custom mixture employing natural-abundance Xe (ca. 26.4%) was sourced from Nova Gas Technologies, North Charleston, SC, USA), the cell is “broken in” with a thermal cycling procedure: first, polarization build-up is typically performed at the highest stable temperature (i.e., below the temperature at which Rb runaway is observed to occur). Polarization build-up continues until steady state is reached, Figure 4c. After reaching these conditions, the SEOP cell is then cooled to room temperature (~25 °C) and maintained there for 30 min to allow for sufficient amount of time to the gas-phase Rb metal to evenly condense on SEOP walls. During the cooling process, the laser power is reduced gradually (down to ~15 W minimum power) until the recorded SEOP cell jacket temperature reaches ~38 °C (i.e., below the Rb melting point); this practice helps to ensure that no significant accumulation of condensed Rb occurs on the front or rear windows of SEOP cell (which can otherwise lead to hot spots).

This process is repeated until we obtain two nearly-identical polarization build-up curves (Figure 4c) under identical conditions; in case additional rounds of temperature cycling are determined to be necessary, we have found this to be an indication of insufficient distribution of Rb metal in the SEOP cell.

Before measuring the HP ^129^Xe build-up rate, the SEOP cell was heated to the desired set point while the laser current was maintained below the nominal operating current of 37.5 A, corresponding to an off-resonance condition, i.e., virtually no photon absorption occurs by Rb. This practice ensures effectively negligible polarization at the start of each build-up experiment (performed at full laser power and on D_1_ resonance corresponding to 37.5 A current), which simplifies the fitting mono-exponential build-up function to y = %*P*_Xe_*(1-exp(-x/*T*_b_), where *T*_b_ is the build-up time constant (mins), x is time (mins), and %*P*_Xe_ is the maximum *P*_Xe_ at t → ∞ (note the fitting function has two variables as y(0) = 0), described in detail in previous work [67].

During hyperpolarization build-up experiments, NMR signals from the SEOP cell were acquired at six-minute intervals (or more frequently if needed) to calculate *P*_Xe_ using Equation (1),

(1) $$P_{\mathrm{Xe}}=P_{H}\left(\frac{C_{H}}{C_{\mathrm{Xe}}}\cdot \frac{\gamma_{H}}{\gamma_{\mathrm{Xe}}}\cdot \frac{S_{\mathrm{Xe}}}{S_{H}}\right)*Corr\left(T_{2}^{*}\right)$$

where *P*_H_ is the thermal polarization of protons and ^129^Xe at 40.8 kHz—equilibrium thermal polarization values are the same for both ^1^H and ^129^Xe when utilizing this detection frequency. *C*_H_ and *C*_Xe_ are proton and ^129^Xe concentrations, γ_H_ and γ_Xe_ are ^1^H and ^129^Xe gyromagnetic ratios, and *S*_H_ and *S*_Xe_ are signal peak integral values of ^1^H and ^129^Xe nuclei, respectively, obtained at an RF pulse length of 150 μs and at a pulse amplitude of -36 dB for ^1^H and -25.3 dB for ^129^Xe, corresponding to ~30 mW and 360 mW, respectively). Because of the difference in the rate of transverse signal decay *T*_2_^*^ for ^129^Xe and ^1^H, a correction factor, *Corr*(*T*_2_^*^), was utilized [60]:

(2) $$Corr\left(T_{2}^{*}\right)=\frac{T_{\mathrm{aq}}}{T_{2}^{*}\mathrm{Xe}}-\frac{T_{\mathrm{aq}}}{T_{2}^{*}H}$$

Here, *T*_aq_ is the pre-acquisition delay time to mitigate the ring-down effect of the RF coil (3 ms in our experiments), and *T*_2_^*^Xe and *T*_2_^*^H are the observed *T*_2_* spin relaxation time constants in ms for ^129^Xe and ^1^H, respectively. At the end of each build-up experiment, an exponential curve fit of ^129^Xe polarization build-up over time was performed using Equation (3) via automated MATLAB processing software described previously [67]:

(3) $$P_{\mathrm{Xe}}\left(t\right)=P_{\max}[1-\exp\left(-\gamma_{\mathrm{SEOP}}\left(t\right))\right]+P_{\mathrm{Xe}}\left(0\right)$$

At any time, *t* (in seconds) after commencement of polarization, the ^129^Xe polarization is determined as a function of the maximum achievable polarization (*P*_max_) at steady state and the polarization build-up rate (γ_SEOP_). *P*_Xe_ at the start of the build-up experiment (i.e., *t* = 0) is negligible because of high-flip angle “crushing” RF pulses (at least 1000 pulses of ~900 μs duration each, applied over the course of 300 s with repetition time of ~0.3 s) are applied before the start of a new polarization build-up experiment.

The polarization build-up rate (γ_SEOP_) determines how quickly steady-state conditions are reached,

(4) $$\gamma_{\mathrm{SEOP}}=\frac{1}{T_{b}}=\gamma_{\mathrm{SE}}+\Gamma_{\mathrm{Xe}}={\;\gamma }_{\mathrm{SE}}+\frac{1}{T_{1}}$$

where *T*_b_ is the polarization build-up time constant, γ_SE_ is the spin-exchange rate (min^−1^) between Rb electrons and ^129^Xe nuclei, Γ_Xe_ is the rate of spin destruction (min^−1^) for ^129^Xe nuclear spins, and *T*_1_ is the longitudinal spin relaxation time constant (min). γ_SEOP_ is obtained from the mono-exponential data fitting of the build-up experiment (e.g., Figure 4c), and *T*_1_ is obtained from the mono-exponential data fitting of HP ^129^Xe relaxation, monitored by small-angle RF excitation pulses, i.e., *T*_1_ decay curve, Figure 4d.

Five NIR spectroscopic measurements (each) were collected under steady-state conditions at the end of each build-up experiment without changing cell temperature or laser power. These spectra were acquired under two different configurations, with the B_0_ magnetic field either powered on (I_Xe_ = 3.08 A) or off (I_Xe_ = 0 A). From these signal-averaged NIR spectra and using Equation (5), we can estimate the Rb polarization, *P*_Rb_ [32],

(5) $$\left|P_{\mathrm{Rb}}\left|=\right|\frac{ln\left(I_{\mathrm{hot}}\right)-ln\left(I_{\mathrm{cold}}\right)}{ln\left(I_{0}\right)-ln(I_{\mathrm{cold}})}\right|$$

where I_hot_ and I_0_ represent the peak area integrals of the pump laser transmission spectra acquired at the temperature where SEOP is performed, with homogeneous *B*_0_ magnetic field powered on and off, respectively. I_cold_ represents the peak area integral of the pump laser transmission spectra acquired under conditions of no optical pumping at room temperature with the magnetic field powered on, as described above. These calculations were performed for each SEOP cell temperature investigated.

At the conclusion of each day of experimentation, an overnight *T*_1_ relaxation measurement was performed to determine the ^129^Xe polarization decay rate (e.g., Figure 4d). From steady-state polarization conditions, the SEOP cell was cooled down to room temperature (~25 °C) by turning on all ten chassis exhaust fans (six already having been on during the SEOP process). While cooling down the SEOP cell, the laser current was maintained as follows. The laser amperage was set to 37.5 A (max power) at the Rb D_1_ resonance frequency at the temperature above 50 °C to minimize %*P*_Xe_ losses during the cool-down process [59]. Laser power was reduced to ~20 W (corresponding to laser PSU current of 15 A and being off-resonance) between 38–50 °C to minimize Rb deposition on the optical flats. The laser power was reduced to 0 W at the temperature below 38 °C, i.e., below Rb melting point. Only once the SEOP cell was cooled down to below 38 °C and the pump laser was completely powered down to ensure no additional laser heating (or optical pumping) occurs did further NMR spectroscopic acquisitions begin. *T*_1_ relaxation measurements were then performed by acquiring ^129^Xe spectra at 4- or 6- minute intervals using 150 μs RF pulses (unless otherwise noted) with I_Xe_ = 3.08 A. The ^129^Xe in-cell *T*_1_ measurements were not corrected for RF-pulse-associated magnetization losses.

### 2.5. SEOP Temperature Mapping

We utilized a SEOP experiment temperature range of 65 to 95 °C (typically with 5 °C intervals) for performing polarization build-up and acquisition of the ^129^Xe NMR and pump laser NIR spectra. The recorded data were then analyzed and presented in the form of a pseudo-2D plot, which we refer to as temperature map. At SEOP cell temperatures below 65 °C, the Rb vapor density and consequent polarization build-up rate was considered too slow to be useful in a clinical-scale production setting. With the increase in temperature inside the SEOP cell, the Rb vapor density increases exponentially, resulting in an accelerated rate of spin exchange, but also reduced overall %*P*_Rb_ (due to increased cell opacity to the incident laser light).

We performed ‘temperature mapping’ of the SEOP cell across the given temperature range, acquiring ^129^Xe polarization build-up, NIR absorption, and ^129^Xe *T*_1_ relaxation spectroscopic data. In the context of the cell-refill QA study, these temperature-mapping experiments helped to monitor the SEOP process performance under various experimental conditions (and also the change in the key parameters). Furthermore, the finding of an optimal temperature that balances between polarization build-up rate and Rb runaway temperature is essential for consistency in clinical HP ^129^Xe production.

### 2.6. NMR Sampling Frequency

NMR measurements for the calculation of *P*_Xe_ and *T*_1_ were acquired at 6 min intervals for this study unless noted otherwise. This value was chosen to provide enough data points for reliable calculation of both *P*_Xe_ and *T*_1_, while minimizing depolarization of HP ^129^Xe gas. A comparison of NMR build-up and relaxation experiments performed at different NMR pulse acquisition frequencies can be found in Figure S6 (SI).

### 2.7. SEOP Cell Reloading QA Study Design

The QA study to test SEOP cell longevity with respect to gas reloading was performed to demonstrate the performance and reproducibility of the GEN-3 ^129^Xe hyperpolarizer to produce highly-polarized, high-density HP ^129^Xe gas mixtures in the batch-mode configuration. Before filling the SEOP cell with a Xe/N_2_ gas mixture (2000 Torr total pressure with 50% Xe and 50% N_2_), the gas-handling manifold inlet line was evacuated to a pressure of 10 mTorr followed by ultra-high-purity (UHP, >99.999%) N_2_ purging (3 cycles). UHP N_2_ gas was obtained from Airgas in Detroit, MI, USA. This procedure reduced the probability of any significant presence of unwanted impurities (e.g., O_2_ and H_2_O) within the PEEK tubing or inside the SEOP cell that could otherwise result in Rb oxidation. After the completion of temperature mapping with a given Xe/N_2_ gas mixture (see Section 2.5), we performed 99 SEOP cell refills with UHP N_2_. The refills with UHP N_2_ were followed by refill with a Xe/N_2_ gas mixture with SEOP temperature mapping. Note, only 98 UHP N_2_ refills were performed between the first two points in the study. This practice simulated the process of normal SEOP cell evacuation and refilling on a clinical production scale, without the need to use large quantities of comparatively expensive Xe gas [40]. Moreover, this practice reduces the enormous amount of time that would be otherwise required for refilling the cell with a Xe/N_2_ gas mixture every time and performing temperature mapping (1–2 days per temperature map). The overall flowchart describing the SEOP cell reloading QA study process is outlined in Figure 4.

**Figure 5:**
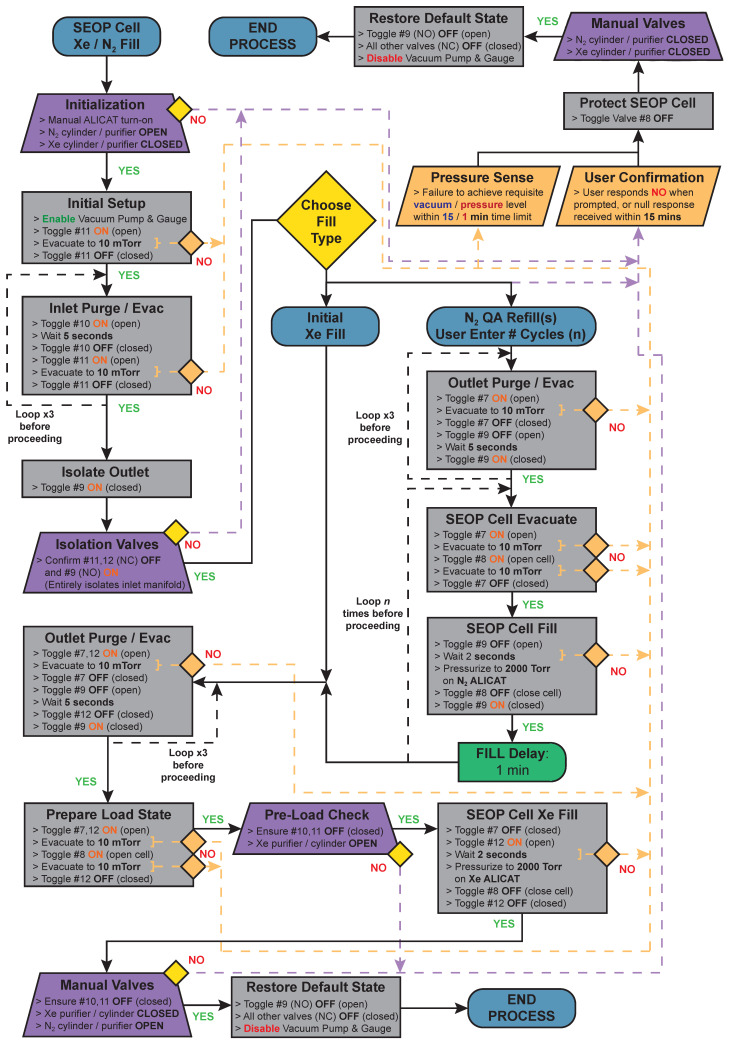
Process flowchart depicting the automated SEOP cell reloading quality assurance (QA) refill sequence coded into the GEN-3 ^129^Xe hyperpolarizer driver module for the purpose of evacuating and refilling SEOP cells. Note, the yellow diamonds indicate condition states. For example, when the user does not provide input within 15 min, the sequence follows the “NO” path. Another example is vacuum level reaching 10 mTorr, which has to be achieved within 15 min, otherwise the sequences defaults to the “NO” path. The timeout duration is 15 min for all yellow diamonds. The valve numbering is provided in Figure 1d.

The sequence for performing automatic vacuum and gas manipulations on the GEN-3 hyperpolarizer is coded into the controller driver module, and is represented schematically in Figure 5. This sequence facilitates the safe and reproducible automated evacuation and refilling of the SEOP cell with either UHP N_2_ or Xe/N_2_ gas mixtures. This process retains some manual checks that will ensure the user is performing the correct adjustments (e.g., manual opening and closing of gas cylinders) and will ensure that all the configurations are correct (e.g., valves are correctly open/closed at the correct times). The sequence allows the user to choose between Xe/N_2_ gas mixture or N_2_ SEOP cell refills, and in the case of N_2_ refills, how many cycles to perform. Therefore, a high degree of consistency in the study can be achieved.

## 3. Results and Discussion

### 3.1. SEOP Cell Reloading QA Study

**Figure 6:**
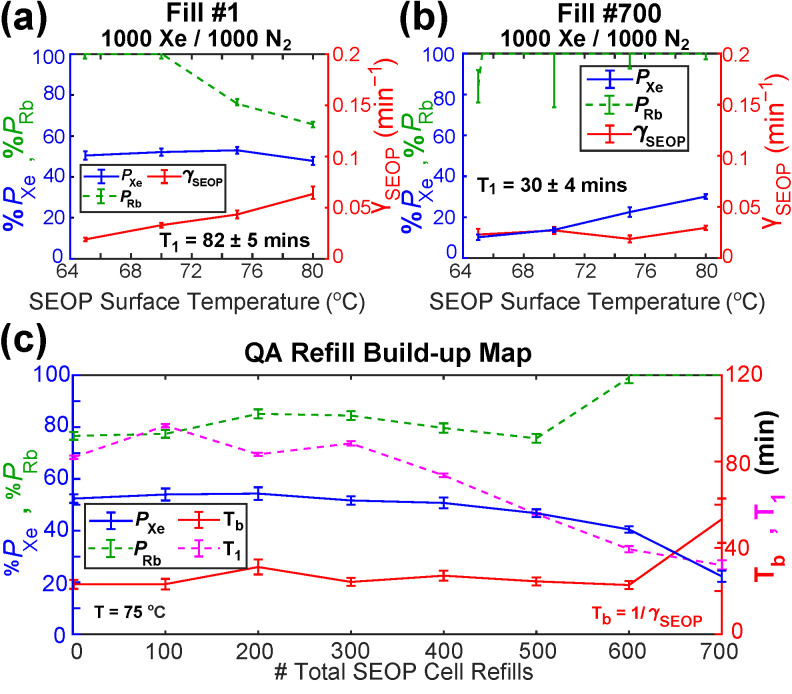
(**a**) Steady-state %*P*_Xe_, γ_SEOP_, and %*P*_Rb_ measurements as a function of cell jacket temperature following fill #1 (the initial 1000 Torr Xe/1000 Torr N_2_ gas mixture fill at the commencement of the QA study). (**b**) Steady-state %*P*_Xe_, γ_SEOP_, and %*P*_Rb_ measurements as a function of SEOP cell surface temperature following fill #700 (the final gas fill of the QA study); note the reduction in %*P*_Xe_ and *T*_1_ values. (**c**) Steady-state %*P*_Xe_, %*P*_Rb_, and *T*_b_ measurements after polarization build-up at a SEOP cell surface temperature of 75 °C, as well as post-build-up *T*_1_ relaxation measurements, plotted versus the number of total gas mixture refills. The solid lines are added to guide the eye.

The GEN-3 hyperpolarizer exhibits robust performance reproducibility. In our previous QA study on the second-generation (GEN-2) ^129^Xe hyperpolarizer [40], we performed an initial temperature-mapping experiment on the first Xe/N_2_ gas mixture fill, and selected a single temperature point (80 °C) from that experiment at which to compare %*P*_Xe_, γ_SEOP_, and %*P*_Rb_ from subsequent SEOP cell refills. In this GEN-3 device study, a temperature map was acquired at SEOP jacket temperatures between 65 and 80 °C (intervals of 5 °C) for all 1000 Torr Xe/1000 Torr N_2_ gas mixture refills, starting with the first fill (Figure 6a), then every 100th subsequent fill to a maximum of 700 (Figure 6b). Temperature-dependent build-up maps for the intermediary fills are shown in Figure S1 (SI). The selected temperature range was chosen to provide polarization build-up rates fast enough to ensure a reasonable time-frame for experimentation, while also avoiding unstable Rb runaway regimes at high temperatures.

The studied SEOP cell exhibited a maximum ^129^Xe polarization exceeding 50%, in overall agreement with previous studies employing a GEN-3 ^129^Xe hyperpolarizer device [39,62]. This level of polarization was reasonably consistent across the range of temperatures investigated, with only a slight reduction at the highest temperature of 80 °C, where the increased vapor density of Rb inside the cell likely led to a reduction in optical pumping rates at the rear of the cell—see the significant reduction in %*P*_Rb_ from ~100% to 65% across the investigated temperature range. This was mitigated by high spin-exchange rates, and hence, high hyperpolarization build-up rates (γ_SEOP_ = 0.082 ± 0.011 min^−1^ at 80 °C), equating to a build-up time constant of only ~12 min, Figure 6a.

This temperature-mapping process was repeated seven more times at intervals of 100 total SEOP cell refills, with all refills comprising the same 1000 Torr Xe/1000 Torr N_2_ fraction. Figure 6b displays the results of the last set of experiments for comparison. Of particular note is the drastic reduction in steady-state %*P*_Xe_ compared to Figure 6a, especially at low temperatures (47.5 ± 1.7% dropping to 10.0 ± 1.3% at 65 °C). This effect also manifests as a reduced ^129^Xe *T*_1_ (70.1 ± 0.8 dropping to 32.0 ± 2.3 min). The reduction in maximum achievable ^129^Xe polarization was less pronounced at higher SEOP cell temperatures, however. This result would be consistent with the change of Rb colligative properties affecting the Rb gas-phase density and hence optical pumping rate. This explanation would be consistent with the decreased optical pumping rate γ_SEOP_.

A comparison of ^129^Xe polarization build-up efficiency at a selected SEOP cell temperature (75 °C in this example, all other variables remained constant) as a function of the number of SEOP cell refills is presented in Figure 6c. The key observation from these results is that no noticeable decrease in %*P*_Xe_ was observed for the first 500 cell refills (avg. %*P*_Xe_ = 51.0 with standard deviation σ*_P_*_Xe_ = 3.02%). A noticeable decrease in %*P*_Xe_ was seen in subsequent experiments, but a final polarization value (after completing 700 refills) %*P*_Xe_ = 24.4 ± 2.4% with γ_SEOP_ = 0.019 ± 0.003 min. Although the ^129^Xe hyperpolarization build-up rate was significantly reduced by the final SEOP cell gas mixture refill, across the first 600 refills a high degree of consistency was observed (avg. *T*_b_ = 25.1 min with standard deviation σ*_T_*_b_ = 3.05 min). The HP ^129^Xe *T*_1_ exhibited more variation than other key performance indicators, but was still reasonably consistent across the first 400 SEOP cell gas mixture refills (avg. *T*_1_ = 82.4 min with standard deviation σ_*T*1_ = 10.8 min). The ^129^Xe hyperpolarization build-up rate γ_SEOP_ (=1/*T*_b_) remained consistent throughout the study with the exception of the last refill at 700 cycles, where *T*_b_ has increased—likely reflecting a decrease in the Rb vapor pressure because of partial Rb oxidation as discussed above. Finally, the rubidium polarization %*P*_Rb_ showed similarly negligible change across the first 500 SEOP cell gas mixture refills (avg. %*P*_Rb_ = 77.8% with standard deviation σ*_P_*_Rb_ = 7.4%), only increasing to ~100% for the final 200 refills where Rb oxidization likely became more substantial; a lower Rb density would translate to higher photon to Rb ratio at a given temperature. Thus, while %*P*_Rb_ is higher, lower %*P*_Xe_ is attained at the near-end of the SEOP cell lifetime because of the significantly lower γ_SEOP_ and *T*_1_.

### 3.2. SEOP Cell Operation with Open Stopcock

**Figure 7:**
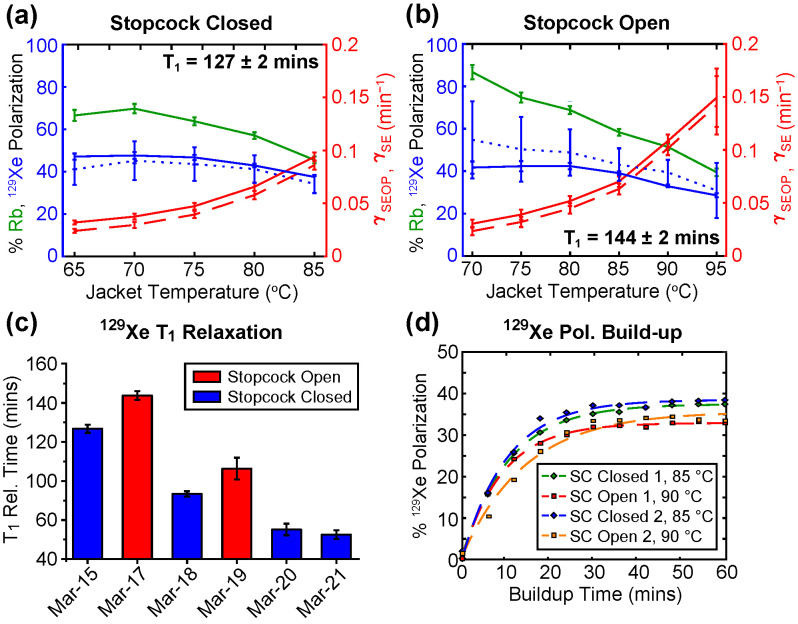
Upper displays (**a**,**b**) describe steady-state %*P*_Xe_, γ_SEOP_, and %*P*_Rb_ measurements as a function of SEOP cell surface temperature in a 1000 Torr Xe/1000 Torr N_2_ gas mixture fill obtained with the SEOP cell stopcock (**a**) closed and (**b**) open. Note, the solid blue line denotes experimental %*P*_Xe_ and dashed blue line denotes theoretically predicated %*P*_Xe(theor)_ based on the experimental values of %*P*_Rb_, γ_SEOP_ and ^129^Xe *T*_1_ as described previously [67] (see SI). Lower displays show comparisons of (**c**) ^129^Xe *T*_1_ values (note the *y*-axis starts at 40 min to better delineates the differences) and (**d**) ^129^Xe polarization build-up curves recorded for the first four experimental points shown in display c—note the consistency in the polarization dynamics for a given cell configuration.

One important limitation of the QA study described above was the fact that the (manually controlled) SEOP cell stopcock was closed during hyperpolarization throughout; however, the stopcock was opened for the duration of each 100 refill cycles, which lasted ~18 h. This requirement for manual operation of the stopcock is undesirable for routine clinical use, and it was employed for this QA study only. Opening and closing the SEOP cell requires the pump laser to be powered down so that the upper chassis of the GEN-3 hyperpolarizer device can safely be opened for access, thus increasing the turnaround time between polarization build-up experiments. To more closely simulate routine clinical-scale production conditions, a series of additional SEOP experiments were performed with the SEOP cell stopcock open to the gas-handling manifold inlet, which is automatically and fully controlled by the solenoid valves and mass flow controllers (Figure S8). Figure 7 shows a pair of SEOP temperature maps obtained with the cell stopcock closed (Figure 7a) or open (Figure 7b), respectively—but under otherwise-identical experimental conditions. A difference in optimal temperature (*T*_OPT_) for performing SEOP likely arises as a consequence of the increased volume and reduced effective Rb vapor density in the stopcock-open configuration compared to the stopcock-closed configuration. From a comparison of Figure 7a,b, it can be inferred that this optimal temperature delta is on the order of ~5–10 °C, with maximum achievable ^129^Xe polarization %*P*_Xe_ values of 47.6 ± 1.6% and 42.4 ± 1.5% observed at SEOP jacket temperatures of 70 and 80 °C in the stopcock-closed and stopcock-open configurations, respectively. In both configurations, a reduction in %*P*_Xe_ is observed at high temperatures and Rb vapor densities; the reduction is more pronounced in the stopcock open configuration, where %*P*_Xe_ drops to 28.5 ± 1.0% at the maximum SEOP jacket temperature of 95 °C—a reduction by 1.33-fold from *T*_OPT_. However, this loss in maximum achievable %*P*_Xe_ is offset by a significant gain in polarization build-up rate γ_SEOP_ in the stopcock open configuration—peaking at 0.149 ± 0.027 min^−1^ at a SEOP cell temperature of 95 °C, compared to a maximum recorded value of 0.070 ± 0.005 min^−1^ at 85 °C in the stopcock closed configuration. This build-up rate increase is welcomed, because it allows for significant scaling up of production efficiency. ^129^Xe *T*_1_ under both configurations was comparable and appreciably long, i.e., in excess of two hours, Figure 7a,b, respectively.

### 3.3. Preparation and Operation of Fluid Path

The results presented in Section 3.1 clearly indicate that the SEOP cell can be reloaded hundreds of times, when good manufacturing practices (GMPs) are properly followed. As a negative control of our experiments, we also provide examples of device points of failure when GMPs were not followed. Results presented in Figure 7c describe the study of ^129^Xe *T*_1_ relaxation time constant over the course of repeated opening and closing of the SEOP cell stopcock. In each instance before opening the SEOP cell stopcock, the inlet line of the gas-handling manifold underwent three cycles of filling and evacuation with UHP N_2_ with the goal to remove any trace quantities of atmospheric air remaining in the inlet line to prevent Rb oxidation in the SEOP cell. This purge-cycling procedure followed an identical GMP protocol as that used in the SEOP cell refilling procedure used within the QA study in Section 3.1, but with two differences: First, when opening the SEOP cell loaded with a 50/50 Xe/N_2_ gas mixture, the cell was not evacuated; instead, the sequence was aborted so that the SEOP cell would remain open. The second and the most critical difference was failure to follow GMPs with respect to a lack of suppression of air contamination of N_2_ loading line. Specifically, before acquiring data from this experiment, the UHP N_2_ cylinder installed on the GEN-3 hyperpolarizer was replaced: normally, we purge the entire N_2_ fluid path (including N_2_ gas purifier as shown in Figure S8) with ten cycles of vacuum (<5 × 10^−2^ Torr) and UHP N_2_ over the course of at least 1 h. However, no purging of the full gas-handling manifold inlet line and inert gas purifiers with this new UHP N_2_ was performed before performing the described build-up experiments. A steady reduction in recorded *T*_1_ from 126.9 ± 1.5 to 52.9 ± 1.1 min was observed due to the ingress of atmospheric air left over from disconnecting the inlet line to the N_2_ cylinder. Surprisingly, somewhat higher values of *T*_1_ were recorded with the SEOP cell stopcocks being open, with reductions in the *T*_1_ value after closing the SEOP cell stopcock, Figure 7c: the origin of this effect is under study. The step-wise T_1_ reduction was reproducible for each stopcock actuation cycle (Figure 7c). In any case, it is imperative that adequate purge-cycling of all parts of the gas-handling manifold is performed when replacing gas cylinders.

It should also be noted that reproducibility of ^129^Xe polarization build-up was good across the experiments these non-GMP compliant experiments as shown by Figure 7d. The four color-coded build-up curves presented are ascribed to the first four entries in Figure 7c, i.e., in alternating configurations of the SEOP cell stopcock being closed and open. %*P*_Xe_ build-up in the closed configuration was performed at a SEOP jacket temperature of 85 °C, whereas open configuration build-up experiments were conducted at 90 °C. We rationalize these findings as follows: when ^129^Xe *T*_1_ is relatively good (i.e., well over 1h), the 1/*T*_1_ term in Eq. 4 is effectively negligible, and therefore, has no substantial impact of the build-up curve parameters. As a result, *T*_1_ measurements can provide an early indication of SEOP cell deterioration before the deleterious effects can be seen on the build-up curves. A representative comparison example of *T*_1_ relaxation processes in an open- and closed-stopcock configuration SEOP cell following ten full-manifold UHP N_2_ purge-cycles is presented in Figure S5, showing no substantial *T*_1_ decrease.

In a separate experiment with a different SEOP cell, we have studied the SEOP open-stopcock configuration to investigate the ^129^Xe *T*_1_ as an indicator on overall SEOP cell “health”, Figure S3. The ^129^Xe *T*_1_ was measured daily (with the exception of one weekend), and the results clearly indicate slow but steady decrease in *T*_1_ from ~139 ± 2 min to ~87 ± 2 min at the end of an 8-day-long study. We rationalize these findings as the result of a slow leak of atmospheric impurities into the part of the PEEK manifold that remained open to the SEOP cell (see also photo of this section in Figure S10); these minor leaks can lead to Rb oxidation over time. Although the SEOP cell after 8 days can still be employed for obtaining highly polarized ^129^Xe (because %*P*_Xe_ is sufficiently high), such SEOP cell deterioration is concerning in the context of long-term use. Therefore, we conclude that once the SEOP experiments are finished for the day, the SEOP cell must be closed for overnight- or longer-term storage to extend the useable lifetime of the SEOP cell for HP ^129^Xe production.

### 3.4. Effect of Rb Distribution in SEOP Cells

As described in the Materials and Methods section above, each SEOP cell studied underwent a robust Rb distribution procedure during preparation for this series of experiments. An additional set of experiments was performed with inadequate distribution (judged by the lack of Rb metal layer “banding” in the SEOP cell—see comparative photos in Figure S4a,b, respectively) of the loaded fresh Rb metal droplet in order to observe the effects on HP ^129^Xe contrast agent production efficiency. The results presented in Figures S5 and S3 show two clear trends: (1) substantial temperature cycling of the inadequately distributed SEOP cell is required; and (2) %*P*_Xe_ levels can be overall similar to those of a cell with well-distributed Rb, albeit with a longer polarization build-up time: see comparison of maps in Figure 6a, Figures S3 and S5a, respectively.

### 3.5. Limitations of Current Design and Future Work

In our original design of the aluminum SEOP cell jacket [39], the ends of the SEOP cell (ca. ~1 inch of the front and rear portions of the cylinder) were not enclosed by the aluminum jacket due to the slightly larger SEOP cell diameter at the ends (where the optical flats are fused with the glass tube, as a consequence of the glass welding process). Here, we have employed three jacket extenders (two 42 mm long pieces in the front and one 38 mm long piece on the back, see Figure S10 for details) to improve the SEOP cell coverage by the variable-temperature aluminum jacket. Our rationale was to improve overall thermal management of the SEOP cell. To our surprise, the results of SEOP cell temperature mapping with and without these jacket extensions (Figure S12) demonstrate no substantial difference between the two jacket configurations. We rationalize our findings in terms of the following hypothesis: the circulating air inside the polarizer chassis removes a substantial amount of heat from the SEOP cell, causing larger-than-anticipated temperature gradients across the SEOP cell jacket (and by extension, the SEOP cell), with the SEOP cell ends being much cooler than anticipated—as a result, the addition of extenders does not substantially improve the SEOP cell coverage by the hot SEOP jacket during the hyperpolarization process (we note that in the current design, the temperature is sensed only in the center of the SEOP cell jacket). We aim to perform further studies to investigate this finding, with the overall goal of improving thermal management of the SEOP cell and improving efficiency of the ^129^Xe SEOP process.

## 4. Conclusions

We performed a pilot QA study of a GEN-3 batch-mode clinical-scale automated ^129^Xe hyperpolarizer. Production of good %*P*_Xe_ levels of ~50% has been demonstrated with high reproducibility of hyperpolarizer performance in a clinical-scale ^129^Xe contrast agent production setting, using high-Xe densities (50% Xe fraction in ~2.6 atm total pressure) and rapid temperature ramping enabled by an aluminum heating jacket surrounding the SEOP cell. ^129^Xe hyperpolarization was performed over the course of 700 gas-loading cycles, simulating long-term HP contrast agent production. Good reproducibility was observed over the first 500 SEOP cell refills. Our results demonstrate a more than 2-fold increase in build-up rate (*T*_b_ of ~26 min vs. ~53 min—note that even faster build-up rates are demonstrated at the expense of slightly lower %*P*_Xe_, Figure 5) relative to previously demonstrated results in a comparable QA study on a second-generation (GEN-2) ^129^Xe hyperpolarizer device [40], with only a minor reduction in maximum achievable %*P*_Xe_. Additionally, we investigated the effects of varying SEOP jacket temperatures, distribution of the Rb metal (as a film across the cell surface), preparation and operation of the fluid path with ultra-high-purity N_2_, when running in open- and closed-cell configurations in the context of the GEN-3 hyperpolarizer installation, performance optimization, and maintenance to consistently produce high ^129^Xe polarization values. We attribute lower than expected %*P*_Xe_ in the GEN-3 design versus the GEN-2 design (~51% vs. ~71%, respectively) [40] in part due to some potential limitations of the current jacket design—work is in progress to address these limitations. The results presented further demonstrate the significant potential for the HP ^129^Xe contrast agent in imaging and bio-sensing applications on a clinical-scale.

## Data Availability

MATLAB-based software for automated NIR and NMR data processing is available for download in Reference #67.

## Supplementary Materials

The following are available online, Figure S1: Summary figure displaying all temperature-dependent ^129^Xe polarization build-up maps for each of the six intermediate (100–600) Xe/N_2_ gas mixture SEOP cell refills performed in the QA study. *T*_1_ values describing the ^129^Xe relaxation process immediately after concluding each temperature-mapping experiment are included for reference. Note the solid blue line denotes experimental %*P*_Xe_ and dashed blue line denotes theoretically predicated %*P*_Xe(theor)_ based on the experimental values of %*P*_Rb_, γ_SEOP_ and ^129^Xe *T*_1_. Figure S2: Comparison of ^129^Xe polarization build-up curves under minor modifications to device configuration and operation. Displays (a,b) compare ^129^Xe polarization build-up at 70 °C with the use of a magnetic and non-magnetic thermocouple on the aluminium heating jacket surface for SEOP cell temperature control. Displays (c,d) compare build-up at 75 °C using both the standard heating jacket described in Ref. [39], and an extended variant covering the entire length of the SEOP cell. Displays (e,f,g) compare build-up at 75 °C with 3, 6 and all 10 external chassis fans enabled, respectively, for the purpose of maintaining thermal stability. Figure S3: Steady-state %*P*_Xe_, γ_SEOP_, and %*P*_Rb_ measurements as a function of SEOP cell surface temperature following polarization build-up in a 1000 Torr Xe/1000 Torr N_2_ gas mixture fill under (a) SEOP cell stopcocks in the closed configuration and (b) SEOP cell stopcocks in the open configuration. (c) Polarization build-up under identical conditions in a 1000 Torr Xe/1000 Torr N_2_ gas mixture test-retest scenario. (d) Reduction of *T*_1_ relaxation time constant over time in the SEOP cell with open-stopcock configuration (note the y-axis starts at 60 mins to better delineates the differences), when gas handling manifold inlet line purge-cycling with UHP N_2_ was not performed. Displays (e) and (f) show the difference between polarization build-up dynamics under otherwise-identical conditions when performing insufficient and sufficient temperature cycling (respectively) of moderately-oxidized SEOP cells. Figure S4: (left) Photos of a GEN-3 SEOP cell (a) before, and (b) after undergoing a Rb distribution procedure over half of the SEOP cell. Note the banded silver-colored film along the inner surface of the SEOP cell where Rb vapor has condensed in significant quantities. Virtually complete vaporization of the Rb droplet is possible over longer timescales, with large cold areas and high temperature gradients (e.g., use of liquid N_2_). (right) Annotated photographs of a Rb distribution setup—see Figure S11 for details of the manifold. Figure S5: Steady-state %*P*_Xe_, γ_SEOP_, and %*P*_Rb_ measurements as a function of SEOP cell surface temperature following polarization build-up in a cell with poorly-distributed Rb and a 1000 Torr Xe/1000 Torr N_2_ gas mixture fill under (a) SEOP cell stopcock in the closed configuration and (b) SEOP cell stopcock in the open configuration. Display (c) shows the difference between polarization build-up dynamics under otherwise-identical conditions of the first (non-temperature-cycled) and subsequent (temperature-cycled) experiments. Display (d) compares the reproducibility of polarization build-up dynamics at the maximum stable SEOP cell temperatures for stopcock closed and open configurations (85 and 95 °C, respectively) on subsequent days of polarizer operation. Display (e) shows the variation in *T*_1_ (performed at the end of each day of experimentation) over the course of this portion of the study. Figure S6: Comparison of ^129^Xe polarization build-up and relaxation rates as a function of delay period between NMR acquisitions. The number situated above each pair of curves indicates the time interval between acquisition pulses for that set of data. The first curve of each interval (a,c,e,g,i,k) displays the ^129^Xe polarization build-up curve. The second curve of each pair (b,d,f,h,j,l) depicts the HP ^129^Xe T_1_ relaxation decay curve. Displays (m,n) show further HP ^129^Xe T_1_ relaxation curves acquired when properly purge-cycling the SEOP cell inlet between experiments. Figure S7: Automated process flowchart depicting the Xe/N_2_ gas mixture ejection sequence to a Tedlar bag and subsequent SEOP cell refilling. Purple trapezoids represent manual/user-led confirmations or actions. Grey rectangles are automated processes. Yellow diamonds are decisions the user can choose between or confirm through the GUI. Orange parallelograms represent active sensing and response to parameters observed outside of acceptable range. Figure S8: A representative state of the XeUS GEN-3 hyperpolarizer Graphical User Interface (GUI) while performing SEOP. Note valve #9 is normally open in the de-energized state. Figure S9: ^129^Xe polarization buildup monitored by RF pulse duration of 150 µs with amplified gain of −23.3 dB during the build-up process. Once the steady-state is achieved the amplitude of RF excitation pulse was corrected to −25.3 dB (correct) value. Note the overestimation of *P*_Xe_ by a factor of 1.22-fold due to stronger *B*_1_ strength. Figure S10: Photographs of the aluminum jacket extenders employed for the SEOP cell. Figure S11: Schematic Diagram of SEOP Cell “Cleaner” and Rb distribution setup (a.k.a. the Rb “spreader”). Figure S12: Comparison of ^129^Xe polarization build-up at different temperatures and temperature map with extended jacket (a,c,e,g,i,k) and without the extended jacket (b,d,f,h,j,l).

## Sample Availability

Samples of the compounds are not available from the authors.
