# Supplementary material for: Pilot Quality-Assurance Study of a Third-Generation Batch-Mode Clinical-Scale Automated Xenon-129 Hyperpolarizer
Source: Molecules. 2022 Feb 16;27(4):1327. doi: 10.3390/molecules27041327 (PMC8879294; doi:10.3390/molecules27041327)
Supplement: Supplementary file 1 [file molecules-27-01327-s001.zip › molecules-1550315- SI_v8.pdf]

SUPPORTING INFORMATION

for

Pilot Quality-Assurance Study of Third-Generation Batch-Mode Clinical-Scale Automated Xenon-129 Hyperpolarizer

Jonathan R. Birchall [1,*,†], Md Raduanul H. Chowdhury [1,†], Panayiotis Nikolaou [2], Yuri A. Chekmenev [2], Anton Shcherbakov [3,4], Michael J. Barlow [5], Boyd M. Goodson [6] and Eduard Y. Chekmenev [1,7,*]

[1] Department of Chemistry, Integrative Biosciences (Ibio), Karmanos Cancer Institute (KCI), Wayne State University, Detroit, MI 48202, USA; raduanul@wayne.edu

[2] XeUS Technologies Ltd., Nicosia 2312, Cyprus; peternikolaou78@gmail.com (P.N.); yura_chekmenev@mail.ru (Y.A.C.)

[3] Smart-A, 614000 Perm, Russia; xbister@gmail.com

[4] Custom Medical Systems (CMS) Ltd., Nicosia 2312, Cyprus

[5] Sir Peter Mansfield Imaging Centre, University of Nottingham, Nottingham NG7 2RD, UK; michaelj.barlow@me.com

[6] Department of Chemistry and Biochemistry, Materials Technology Center, Southern Illinois University, Carbondale, IL 62901, USA; bgoodson@chem.siu.edu

[7] Russian Academy of Sciences, Leninskiy Prospekt 14, 119991 Moscow, Russia

* Correspondence: jonathan.birchall@outlook.com (J.R.B.); chekmenevlab@gmail.com (E.Y.C.)

† These authors contributed equally to this work.

Table of Contents

1) Introduction to Supporting Information S-2
2) Intermediate QA Refill Temperature Mapping S-3
3) Assessment of Effects of GEN-3 Operation on P_{Xe} and γ_{SEOP} S-5
4) Effect of SEOP Cell Inlet Purge-cycling on Long-term SEOP Cell "Health" S-6
5) Effect of Rb Distribution on SEOP Process Efficiency and Reproducibility S-8
6) Assessment of How Frequently NMR Acquisition is Performed on P_{Xe}, γ_{SEOP} and T_1 .... S-10
7) HP ^{129}Xe Contrast Agent Ejection and SEOP Cell Refilling S-12
8) XeUS GEN-3 ^{129}Xe Hyperpolarizer Graphical User Interface (GUI) S-13
9) Effect of RF Pulse Amplitude on "Apparent" P_{Xe} S-13
10) Aluminum Jacket Extenders for the SEOP Cell S-14
11) Schematic of Rb Distribution (a.k.a. "Spreader") Setup S-15
12) References Used in Supporting Information S-17

1) Introduction to Supporting Information

This Supporting Information (SI) document presents supplementary figures that summarize data obtained over the course of the quality-assurance (QA) procedure. The first of these details SEOP cell performance during ^{129}Xe polarization build-up (P_{Xe}, γ_{SEOP}, and P_{Rb}) as a function of SEOP heating jacket temperature, as well as subsequent T_1 measurement for each intermediate Xe/N_2 gas mixture refill performed (shown in Figure S1, summarized in Figure 6 of the main text).

Also included are results from a number of miscellaneous experiments assessing the GEN-3 hyperpolarizer performance and production consistency under slight differences in operation or design. These include varying the number of external chassis fans in operation, the length of the aluminum SEOP cell heating jacket, and the design of the heating jacket thermocouple (displayed in Figure S2), as well as the frequency of NMR spectral acquisition during both ^{129}Xe polarization build-up and relaxation (Figure S3).

A series of experiments corresponding to the ones described in Figure 7 of the main text, where purging and evacuation of the SEOP cell inlet with N_2 (before opening and closing the SEOP cell stopcock) were not performed, is shown in Figure S3. These results demonstrate further the importance of maintaining an inert environment upstream of the SEOP cell when attempting to polarize xenon consistently on a clinical scale, and the negative effect that even very small atmospheric contributions can have over time.

Lastly, an automated process flowchart for the process of HP Xe/N_2 gas mixture ejection into a Tedlar bag, and subsequent refilling of the SEOP cell with new Xe/N_2 gas mixture, is presented in Figure S4.

2) Intermediate QA Refill Temperature Mapping

To provide further details of the HP ^{129}Xe production consistency of the GEN-3 device, temperature-dependent build-up maps from intermediate gas-refill / SEOP cycles of the longitudinal QA study are presented in Figure S1, ranging from the first intermediate map (acquired after 100 total SEOP cell refills, display a) to the last intermediate map (acquired after 600 total SEOP cell refills, display f). It can be seen that early on in SEOP cell lifetime, high levels of ^{129}Xe polarization can be achieved at comparatively low SEOP cell temperatures, owing to the higher representative Rb vapor densities and optical pumping rates accessible when Rb purity is high. Under these conditions, high SEOP cell temperatures can actually be detrimental to hyperpolarization efforts, as the increasing Rb vapor density leads to optical occlusion (and often, uncontrollable thermal runaway) at the font of the cell, with little to no laser light reaching the rear of the cell. Conversely, in aged cells where Rb has begun to oxidize, the effective vapor pressure is reduced compared to that expected at a given temperature. This reduction in Rb vapor density results in a significant decrease in %P_{Xe}, particularly at low SEOP cell temperatures; on the other hand, the lower Rb vapor pressure in oxidized SEOP cells means that thermal runaway conditions may not be experienced. This effect results in an increase in the "optimal" SEOP cell temperature for efficient ^{129}Xe hyperpolarization over the cell's lifetime. The polarization build-up rate γ_{SEOP} increased roughly linearly with SEOP cell jacket temperature throughout the various stages of the SEOP cell's lifetime. It is also possible to calculate the Rb-^{129}Xe spin-exchange rate γ_{SE} using the following equation [1]:

$$\gamma_{SE} = \gamma_{SEOP} - \frac{1}{T_1} \qquad \text{(S1)}$$

The theoretical ^{129}Xe polarization at steady state can be computed in accord to the following equation [1]:

$$P_{Xe(theor.)} = P_{Rb} \cdot \frac{\gamma_{SE}}{\gamma_{SEOP}} \qquad \text{(S2)}$$

Note that estimates of the theoretical %$P_{Xe(theor)}$ and spin-exchange rate (γ_{SE}) are dependent on the T_1 relaxation time constant, and so may give unreliable or even physically impossible negative value results in cases where γ_{SEOP} and T_1 are both low (see 65 °C results in displays b and f below).

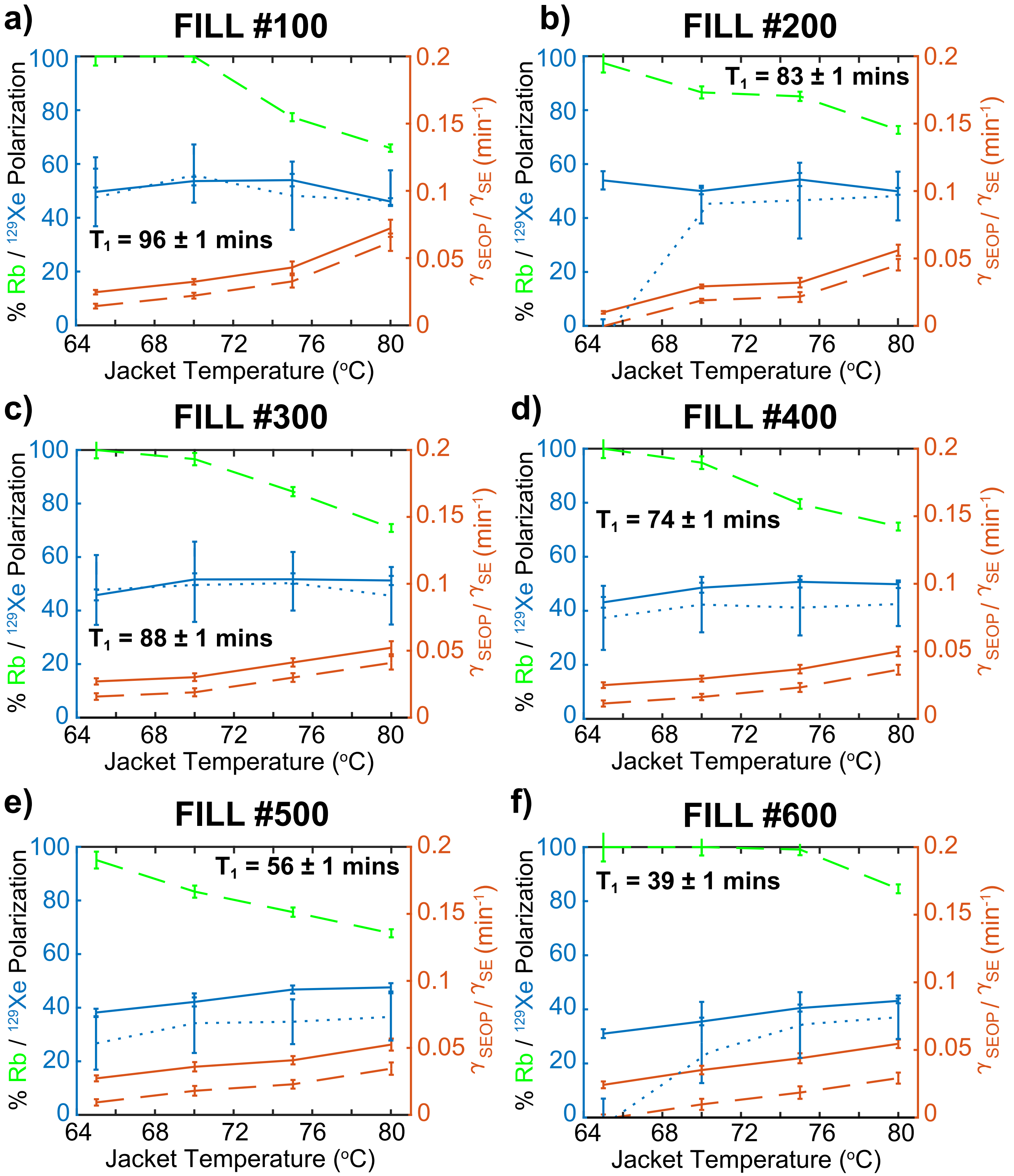

Figure S1: Summary figure displaying all temperature-dependent ^{129}Xe polarization build-up maps for each of the six intermediate (100-600) Xe/N_2 gas mixture SEOP cell refills performed in the QA study. T_1 values describing the ^{129}Xe relaxation process immediately after concluding each temperature-mapping experiment are included for reference. Note the solid blue line denotes experimental $\%P_{Xe}$ and dashed blue line denotes theoretically predicated $\%P_{Xe(theor)}$ based on the experimental values of $\%P_{Rb}$, γ_{SEOP} and ^{129}Xe T_1 as described previously [1] (see Equation S1 and S2 above).

3) Assessment of Effects of GEN-3 Operation on P_{Xe} and γ_{SEOP}

When determining the optimal operational conditions of the GEN-3 ^{129}Xe hyperpolarizer for the QA study, a variety of different device configurations were tested; although some configurational changes were deemed to have only a minor effect (or no effect at all) on HP ^{129}Xe production efficiency, they are included here for completeness. The first comparison concerned the replacement our original metallic SEOP cell thermocouple with a non-magnetic FluxTeq thermocouple, which resulted in a ~4.5% increase in maximum achievable ^{129}Xe polarization at 70 °C (deemed to be within error bar) at a nearly identical polarization build-up rate, γ_{SEOP} (displays a and b), Figure S2a and Figure S2b respectively. One potential explanation is the deleterious effect of magnetic thermocouple on the B_0 field homogeneity in the cell center, where polarization sensing is performed.

The second comparison tests a hypothesis from a previous GEN-3 hyperpolarizer study [2] that extending the aluminum heating jacket to cover the entire length of the SEOP cell would create a more even temperature distribution across the length of the cell and increase %P_{Xe} (or at least, the contributions to %P_{Xe}) within the previously colder front and rear regions of the cell. Unfortunately, in the pair of 75 °C build-up experiments we conducted, no noticeable change was observed (Figure S2c and S2d). There are two alternative ways to rationalize this finding. First (and unlikely), the larger temperature gradients (created at the end of the cell without jacket extenders shown in Figure S10) are hardly detrimental to the SEOP process, or 2) the new additions don't work that well yet. We are currently investigating this question, which was prompted by this pilot QA study, see the main text for more details. Additional data is presented in Figure S12.

Lastly, the number of chassis fans enabled during polarization build-up at 75 °C was varied to determine how SEOP cell jacket temperature oscillation affects the xenon polarization dynamics. Increasing the number of active chassis fans caused greater oscillation in SEOP jacket surface temperature due to the increased heat removal efficiency and the corresponding requirement of the heating elements to draw more power in response. Operating in any of three tested configurations (3-, 6- and 10 fans respectively, Figures S2e-g) resulted in no substantial differences in %P_{Xe} and γ_{SEOP}. We conclude that the device can be operated in a manner suitable to reduce hyperpolarization production cycle: i.e., three fans during SEOP process (to minimize temperature oscillation and reduce the amount and heating and thus power consumption), and ten fans during SEOP cell cool-down process to reduce the production cycle time.

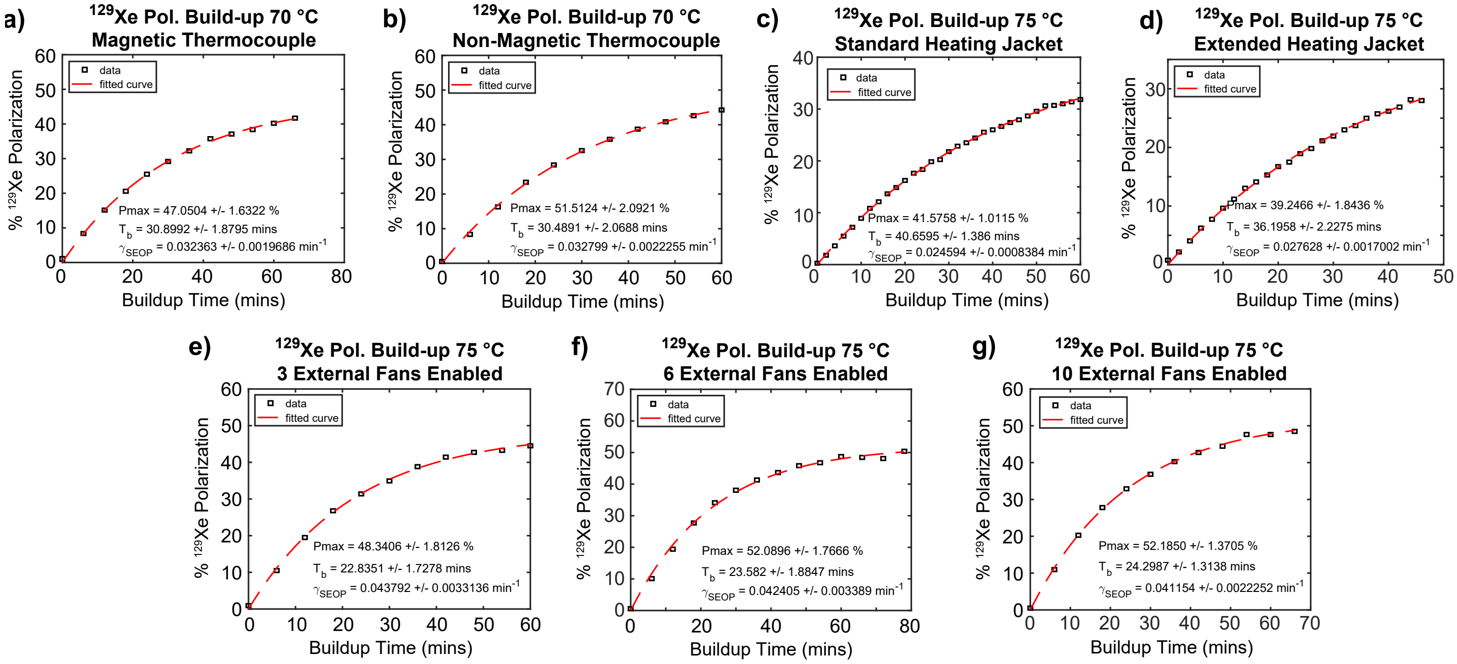

Figure S2: Comparison of ^{129}Xe polarization build-up curves under minor modifications to device configuration and operation. Displays (a,b) compare ^{129}Xe polarization build-up at 70 °C with the use of a magnetic and non-magnetic thermocouple on the aluminium heating jacket surface for SEOP cell temperature control. Displays (c,d) compare build-up at 75 °C using both the standard heating jacket described in Ref. [2], and an extended variant covering the entire length of the SEOP cell. Displays (e,f,g) compare build-up at 75 °C with 3, 6 and all 10 external chassis fans enabled, respectively, for the purpose of maintaining thermal stability.

4) Effect of SEOP Cell Inlet Purge-cycling on Long-term SEOP Cell "Health"

For simulating clinical-scale HP ^{129}Xe contrast agent production, it is desirable to operate the GEN-3 hyperpolarizer in a configuration where the (manually controlled) SEOP cell stopcocks are open, to more easily facilitate ejection of the gas mixture into the Tedlar bag storage vessel and subsequently refill the cell with new Xe/N_2 gas mixture. Operating in the stopcock-open configuration (Figures S3b) shows that the optimal polarization build-up rate γ_{SEOP} is achieved at a temperature delta in excess of 10 °C compared to the stopcock-closed configuration (Figures S3b) albeit with substantial reduction in maximum achievable $\%P_{Xe}$. Effective rubidium polarization $\%P_{Rb}$ is also reduced in the stopcock-open configuration. We are currently investigating the reasons of the differences in SEOP dynamics between the stopcock-open and stopcock-closed configurations to provide an explanation beyond the observational results presented in Figure S3. Despite the reductions in $\%P_{Xe}$ and $\%P_{Rb}$, the significant increase in γ_{SEOP} results in an increase in the Dose Equivalent (DE) production rate (as expected), which satisfies the requirements of clinical-scale production. Reproducibility of these results is demonstrated in a test-retest experiment (display c), with near-identical $\%P_{Xe}$ and polarization build-up time constant T_b values observed across subsequent production cycles.

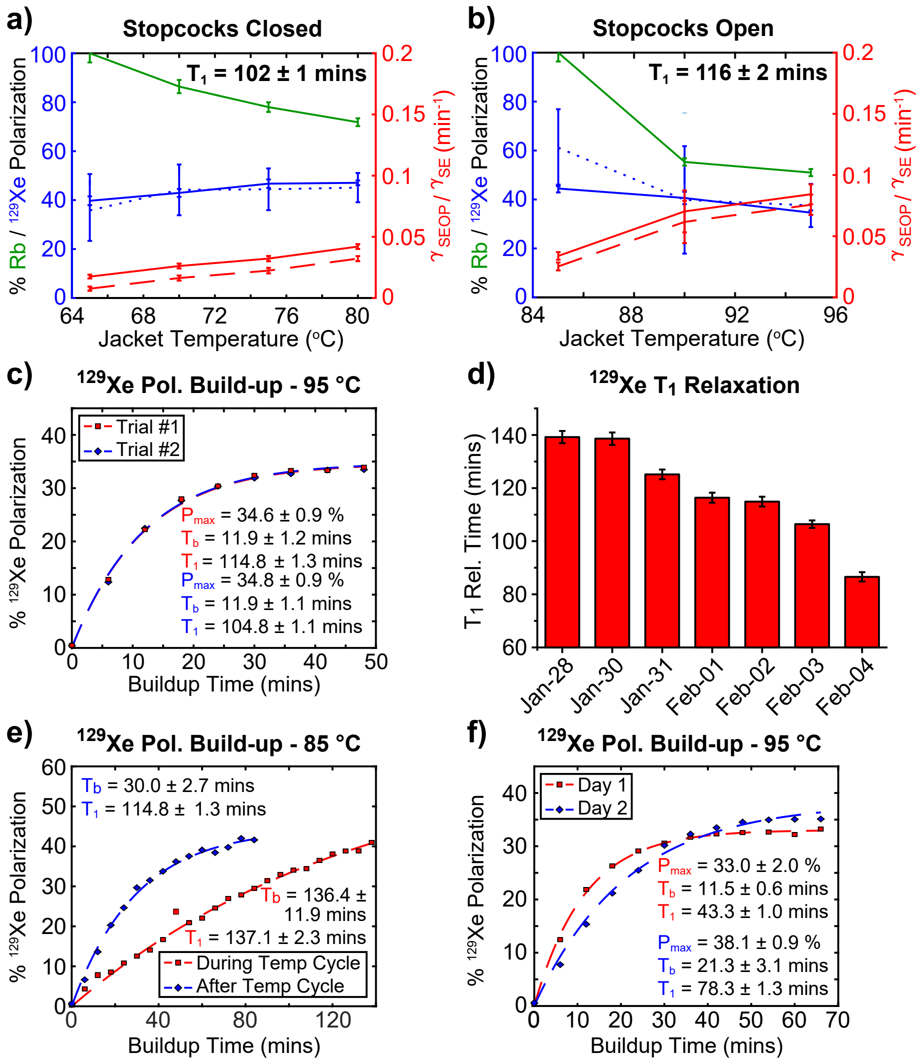

Figure S3: Steady-state $\%P_{Xe}$, γ_{SEOP}, and $\%P_{Rb}$ measurements as a function of SEOP cell surface temperature following polarization build-up in a 1000 Torr Xe / 1000 Torr N_2 gas mixture fill under a) SEOP cell stopcocks in the closed configuration and b) SEOP cell stopcocks in the open configuration. c) Polarization build-up under identical conditions in a 1000 Torr Xe / 1000 Torr N_2 gas mixture test-retest scenario. d) Reduction of T_1 relaxation time constant over time in the SEOP cell with open-stopcock configuration (note the y-axis starts at 60 mins to better delineates the differences), when gas handling manifold inlet line purge-cycling with UHP N_2 was not performed. Displays e) and f) show the difference between polarization

build-up dynamics under otherwise-identical conditions when performing insufficient and sufficient temperature cycling (respectively) of moderately-oxidized SEOP cells.

In our current experimental protocol, the inlet line of the GEN-3 hyperpolarizer gas-handling manifold undergoes three purge-and-evacuate cycles with ultra-high purity (UHP) N_2 before opening the SEOP cell stopcock to minimize the potential for Rb oxidation. As shown in Figure S3d, the longitudinal ^{129}Xe spin-relaxation time constant T_1 was shown to decrease at an unacceptable rate with subsequent days of experimentation using SEOP cells where the stopcocks were maintained in the open configuration. Such Rb oxidation ostensibly occurred because of a large pressure gradient between the SEOP cell (high positive pressure), inlet line (hard vacuum), and the external atmosphere, causing small quantities of atmospheric air to ingress into the inlet line of the manifold over time. Without purge-cycling of the inlet line, these impurities may invariably make their way into the cell when opening the SEOP cell stopcock, leading to Rb oxidation and a gradual decrease in purity and T_1.

In scenarios where inlet line purge-cycling with UHP N_2 was not performed (see the main text discussion of Figure 7, temperature cycling was required to bring fresh, un-oxidized Rb from the bulk liquid to the SEOP cell surface. This process of SEOP cell "regeneration" has been described in more detail during corollary QA studies on a second-generation ^{129}Xe hyperpolarizer [3]. A comparison between ^{129}Xe polarization build-up in temperature-cycled and non-temperature-cycled SEOP cells under otherwise identical conditions is shown in Figure S3e. Note the >4-fold increase in hyperpolarization build-up time constant T_b after performing temperature-cycling. Once the initial temperature cycling of SEOP cell has been performed, the subsequent cell operation becomes more robust—for example, note the similarity of maximum achievable $\%P_{Xe}$ and effective T_b on the second day, to those obtained after temperature-cycling on the first day (Figure S3f).

5) Effect of Rb Distribution on SEOP Process Efficiency and Reproducibility

In addition to purge-cycling of the inlet manifold, we also increase the surface area of the Rb within the SEOP cell via the application of a high-temperature gradient during initial cell preparation. This practice helps to maximize the effective Rb vapor density throughout the cell volume at lower SEOP cell temperatures and thus increase the optical pumping rate. The alkali metal distribution process, briefly discussed in previous work [2], is performed immediately after successful loading of Rb into the SEOP cell, and before installation into the hyperpolarizer and loading of a desired Xe/N_2 gas mixture. The SEOP cell is first placed under vacuum to ~10 mTorr. Upon reaching a pressure of <5 mTorr, the SEOP cell stopcock is sealed following the heating of the Rb droplet to increase the Rb vapor density inside the SEOP cell. This practice ensures the bulk Rb enters the liquid phase, and any trace impurities from the filling or transfer stage that survive the initial evacuation are gettered by the Rb. A temperature gradient is created along the length of the cell via application of dry ice or liquid N_2 to the exterior cell surface. Vaporized Rb then proceeds to condense on the interior of SEOP cell wall in this cold region. By continuing to maintain the temperature gradient, the quantity of Rb condensation on the cell wall will increase, raising the surface area. A visual comparison of a SEOP cell before and after undergoing a Rb distribution procedure is shown in Figure S4 below.

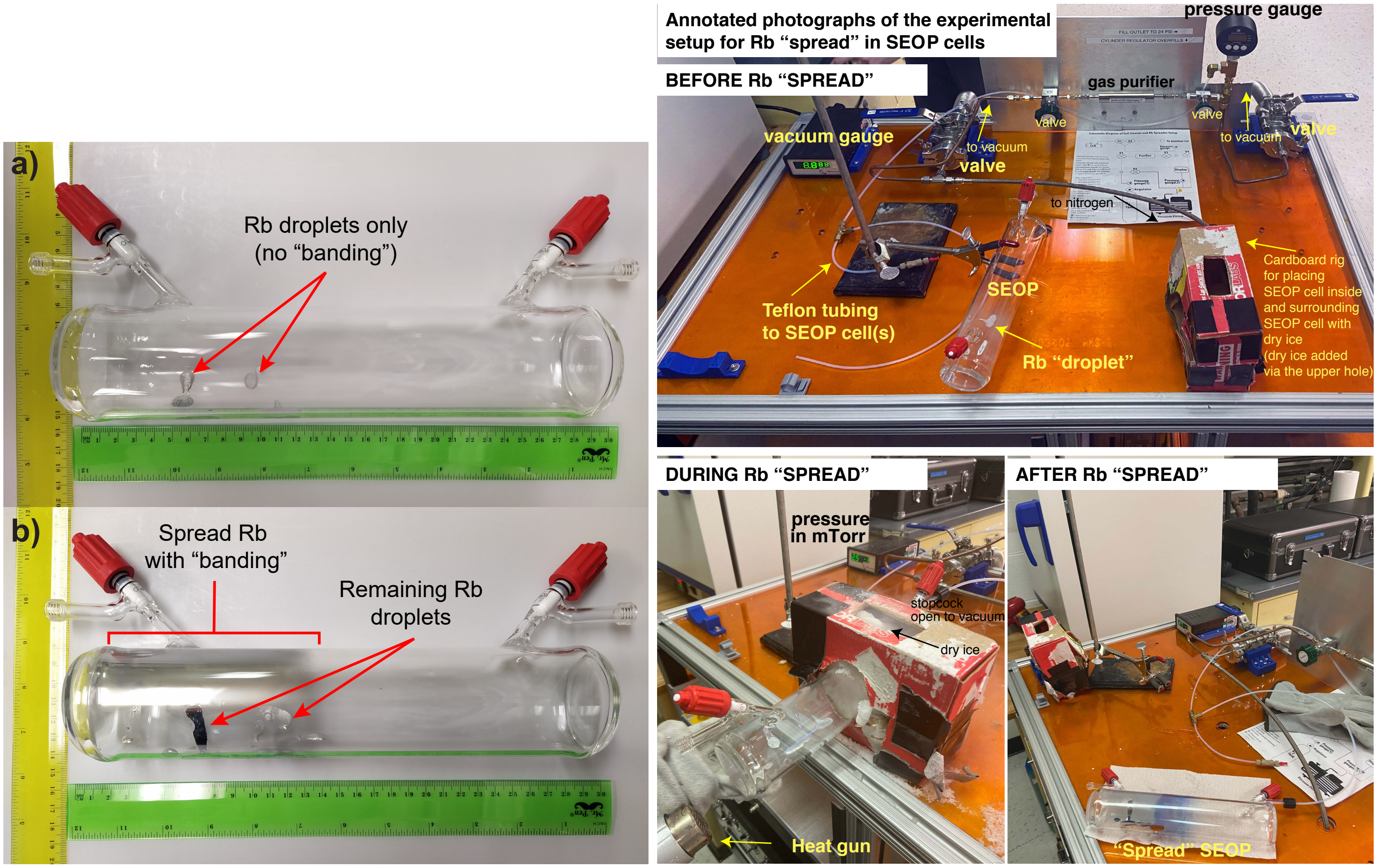

Figure S4: (left) Photos of a GEN-3 SEOP cell a) before, and b) after undergoing a Rb distribution procedure over half of the SEOP cell. Note the banded silver-colored film along the inner surface of the SEOP cell where Rb vapor has condensed in significant quantities. Virtually complete vaporization of the Rb droplet is possible over longer timescales, with large cold areas and high temperature gradients (*e.g.* use of liquid N_2). (right) Annotated photographs of a Rb distribution setup – see Figure S11 for details of the manifold.

In order to assess the degree to which increasing Rb surface area improved GEN-3 hyperpolarizer performance, we performed a pair of temperature-dependent build-up mapping experiments at a range of temperatures with the SEOP cell stopcock both closed and open, respectively, in cells with relatively poor Rb distribution. The results of these investigations are presented in Figure S5. The steady-state ^{129}Xe polarization values and associated build-up and relaxation rates were consistent with previous experiments under both configurations. Unsurprisingly, some amount of temperature cycling is also required to draw

optimal performance out of the SEOP cell with poor Rb distribution (Figure S5c). In this instance, the Rb vapor density is low solely due to the low initial surface area as most of the Rb remains in the bulk liquid. Additionally, the degree of Rb distribution does not appear to have significant impact on production consistency after the initial temperature-cycling process is performed, regardless of whether the SEOP cell stopcock is open or closed (display d). This finding implies that cells with poorly distributed Rb can at least be useful in a clinical-scale production setting where reproducibility between trials is important, albeit with a slight reduction in performance.

A main positive observation of the study involving SEOP cells with relatively undistributed Rb is that of increased T_1 stability over the course of many cycles of opening and closing the SEOP cell stopcock (display e). From an initial value of 97.8±1.1 mins, relaxation effects were actually observed to become less prevalent, eventually reaching 134.2±1.7 mins after 10 different days of experimentation (no T_1 relaxation measurement was performed on March 13th). We hypothesize that ^{129}Xe T_1 increase is due to additional SEOP cell temperature cycling beyond that shown in Figure S5c. Throughout this portion of the study, the GEN-3 hyperpolarizer gas-handling manifold was properly purge-cycled (three alternating cycles of evacuation to 10 mTorr and pressurization to 2000 Torr) with UHP N_2 before each act of opening the SEOP cell stopcock. To conclude when the stopcock is properly opened and closed (*i.e.*, when the upstream manifold is properly purge cycled with UHP inert gas), no T_1 deterioration is seen as expected. This observation is in sharp contrast with the study described in Figure 7 of the main text.

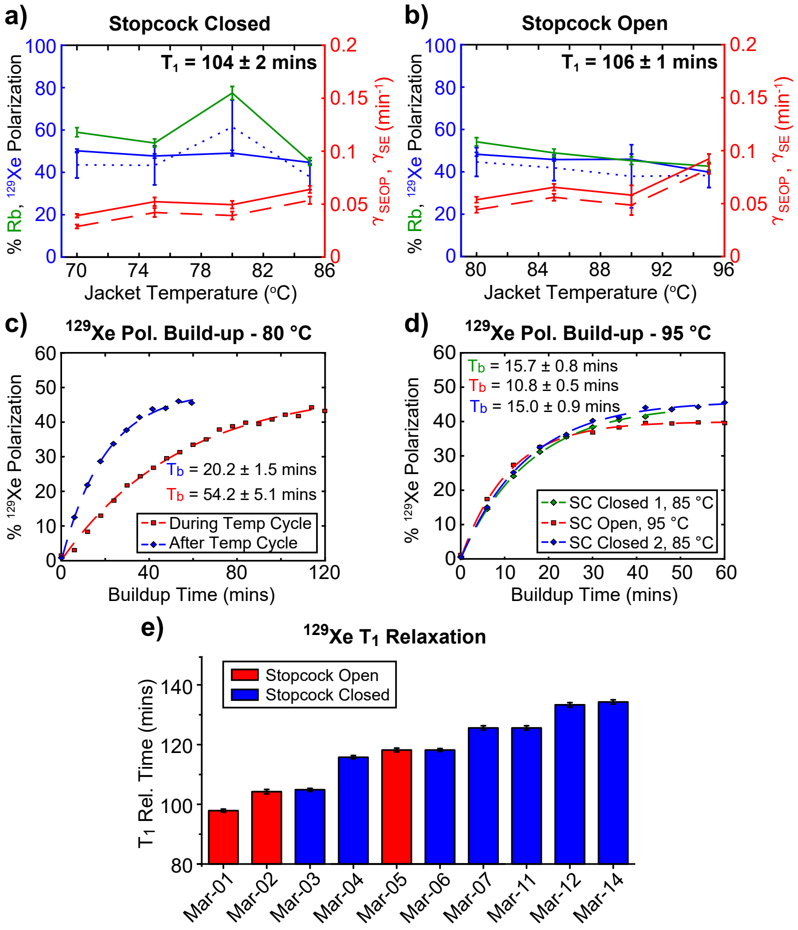

Figure S5: Steady-state $\%P_{Xe}$, γ_{SEOP}, and $\%P_{Rb}$ measurements as a function of SEOP cell surface temperature following polarization build-up in a cell with poorly-distributed Rb and a 1000 Torr Xe / 1000 Torr N_2 gas mixture fill under a) SEOP cell stopcock in the closed configuration and b) SEOP cell stopcock in the open configuration. Display c) shows the difference between polarization build-up dynamics under otherwise-identical conditions of the first (non-temperature-cycled) and subsequent (temperature-cycled) experiments. Display d) compares the reproducibility of polarization build-up dynamics at the maximum stable SEOP cell temperatures for stopcock closed and open configurations (85 and 95 °C, respectively) on subsequent days of polarizer operation. Display e) shows the variation in T_1 (performed at the end of each day of experimentation) over the course of this portion of the study.

6) Assessment of How Frequently NMR Acquisition is Performed on P_{Xe}, γ_{SEOP} and T_1

In addition to determining the optimal RF pulse durations, magnetic solenoid coil B_0 field strengths, and NMR spectrometer B_1 central frequencies for ^{1}H and ^{129}Xe, it was also important to determine a suitable interval of NMR spectral acquisition. A high data acquisition rate improves the reliability of exponential fitting functions and reduces uncertainty in key performance indicators (KPIs) of ^{129}Xe hyperpolarization efficiency, such as $\%P_{Xe}$, γ_{SEOP}, and T_1. However, since hyperpolarization is only recoverable with further application of the SEOP process, each RF pulse performed during NMR spectroscopy acts to depolarize the ^{129}Xe spin system slightly in the active volume of the surface coil. Since diffusion and/or convection occurs throughout the SEOP cell along thermal convection gradients, the depolarized ^{129}Xe spins in the active volume will be replaced with hyperpolarized spins over time from other parts of the cell that do not see depolarizing RF pulsing. When the time interval between RF pulses is appreciably low, depolarized ^{129}Xe spins may not have time to leave the active volume, or be repolarized before entering again at a later time. The combination of these two effects can lead to an *apparent* increase in the measured spin-relaxation (*i.e.*, the decrease in apparent T_1)—and correspondingly, accelerated polarization decay rates—as well as an apparent reduction in $\%P_{Xe}$.

This effect can be readily seen in Figure S6, where the maximum achievable $\%P_{Xe}$ value is lowest at the smallest RF pulse interval of 30 seconds, and increases with increasing RF pulse interval at 1-minute, and plateauing with use of a 2-minute (or greater) interval. When we consider the longitudinal ^{129}Xe spin-relaxation time constant T_1, we again see the shortest relaxation time at the 30 second interval between NMR acquisitions, and an increase with rising acquisition interval up to almost double this value at long acquisition intervals of 6-10 minutes. This observation indicates that estimates of $\%P_{Xe}$ and T_1 are typically under-valued when the rate of RF pulsing is high. We can confirm that this effective reduction in $\%P_{Xe}$ is in fact proportional to the rate of RF pulsing, by considering that the initial ^{129}Xe spin polarization values at the commencement of T_1 decay monitoring are all relatively consistent between trials.

Following this series of experiments, a final pair of T_1 relaxation plots were acquired with six minute intervals for NMR data sampling. The first curve was acquired following build-up at 95 °C in an open-stopcock configuration after performing UHP N_2 purge-cycling of the inlet manifold. The second curve was acquired after closing the stopcock and performing build-up at 80 °C in the closed-stopcock configuration. In both cases, no significant difference observed between the two measurements as theorized from previous experiments shown in Figure S3 and Figure S5.

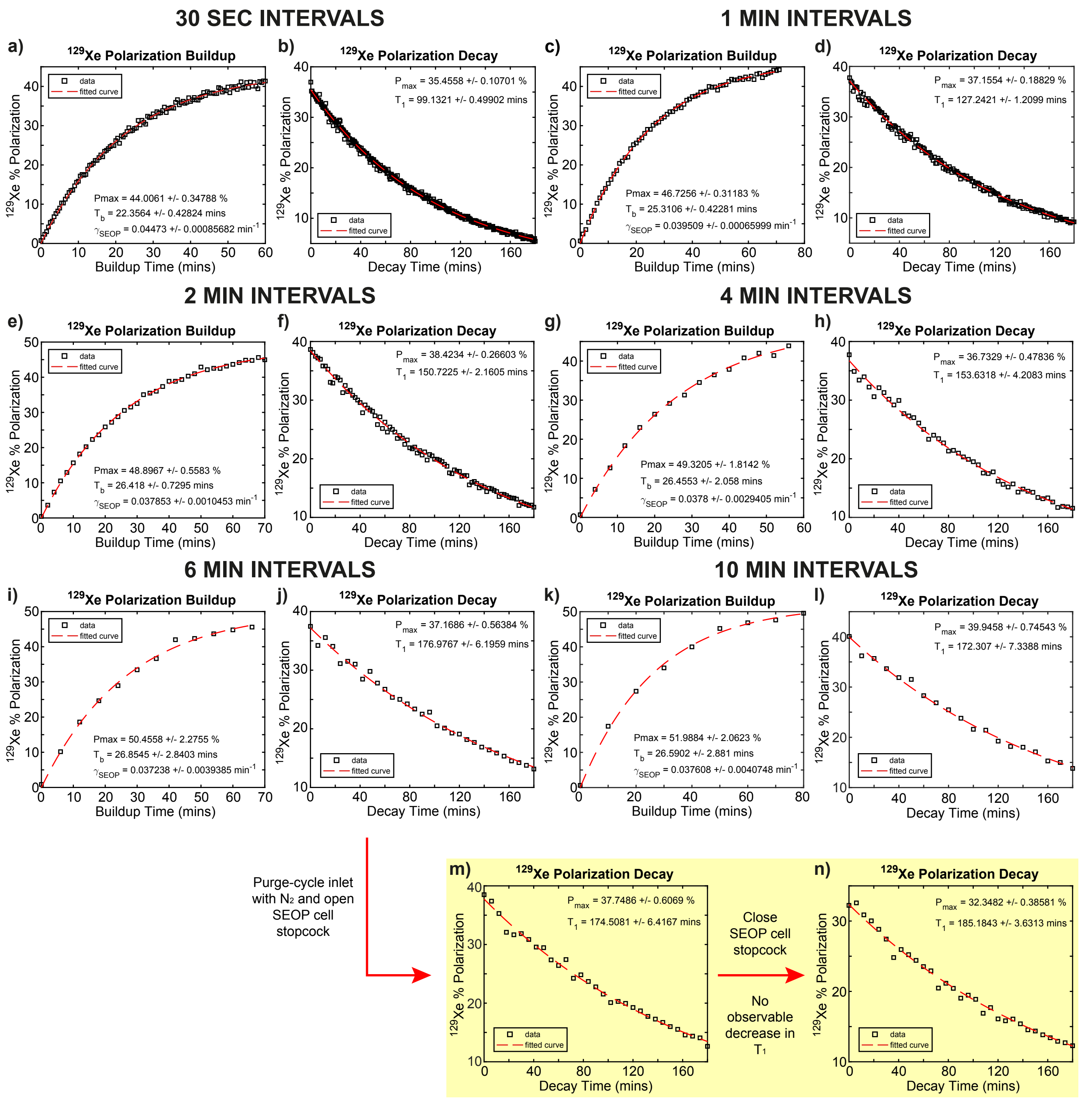

Figure S6: Comparison of ^{129}Xe polarization build-up and relaxation rates as a function of delay period between NMR acquisitions. The number situated above each pair of curves indicates the time interval between acquisition pulses for that set of data. The first curve of each interval (a,c,e,g,i and k) displays the ^{129}Xe polarization build-up curve. The second curve of each pair (b,d,f,h,j and l) depicts the HP ^{129}Xe T_1 relaxation decay curve. Displays (m,n) show further HP ^{129}Xe T_1 relaxation curves acquired when properly purge-cycling the SEOP cell inlet between experiments.

7) HP ^{129}Xe Contrast Agent Ejection and SEOP Cell Refilling

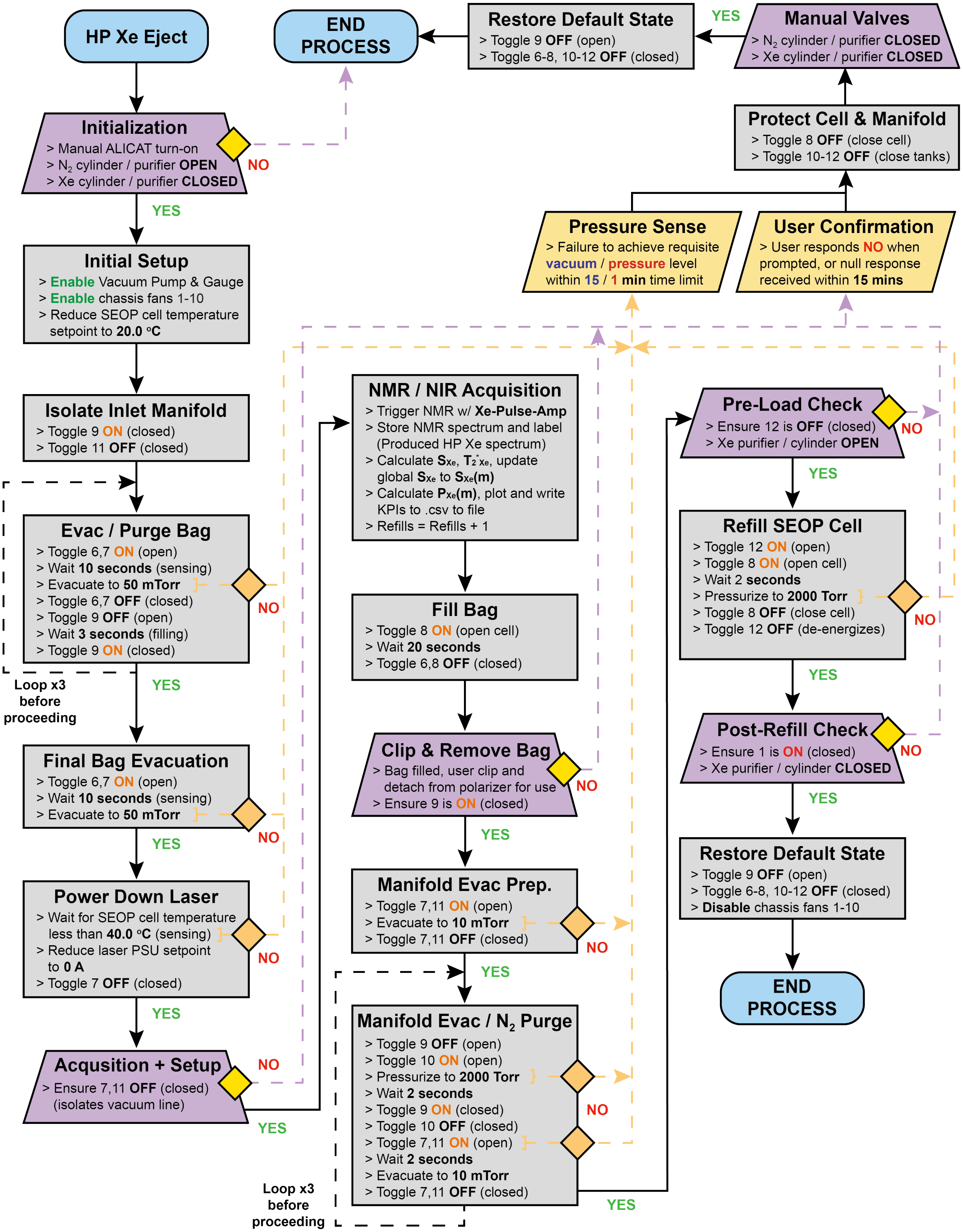

Figure S7: Automated process flowchart depicting the Xe/N_2 gas mixture ejection sequence to a Tedlar bag and subsequent SEOP cell refilling. Purple trapezoids represent manual / user-led confirmations or actions. Grey rectangles are automated processes. Yellow diamonds are decisions the user can choose between or confirm through the GUI. Orange parallelograms represent active sensing and response to parameters observed outside of acceptable range.

8) XeUS GEN-3 ^{129}Xe Hyperpolarizer Graphical User Interface (GUI)

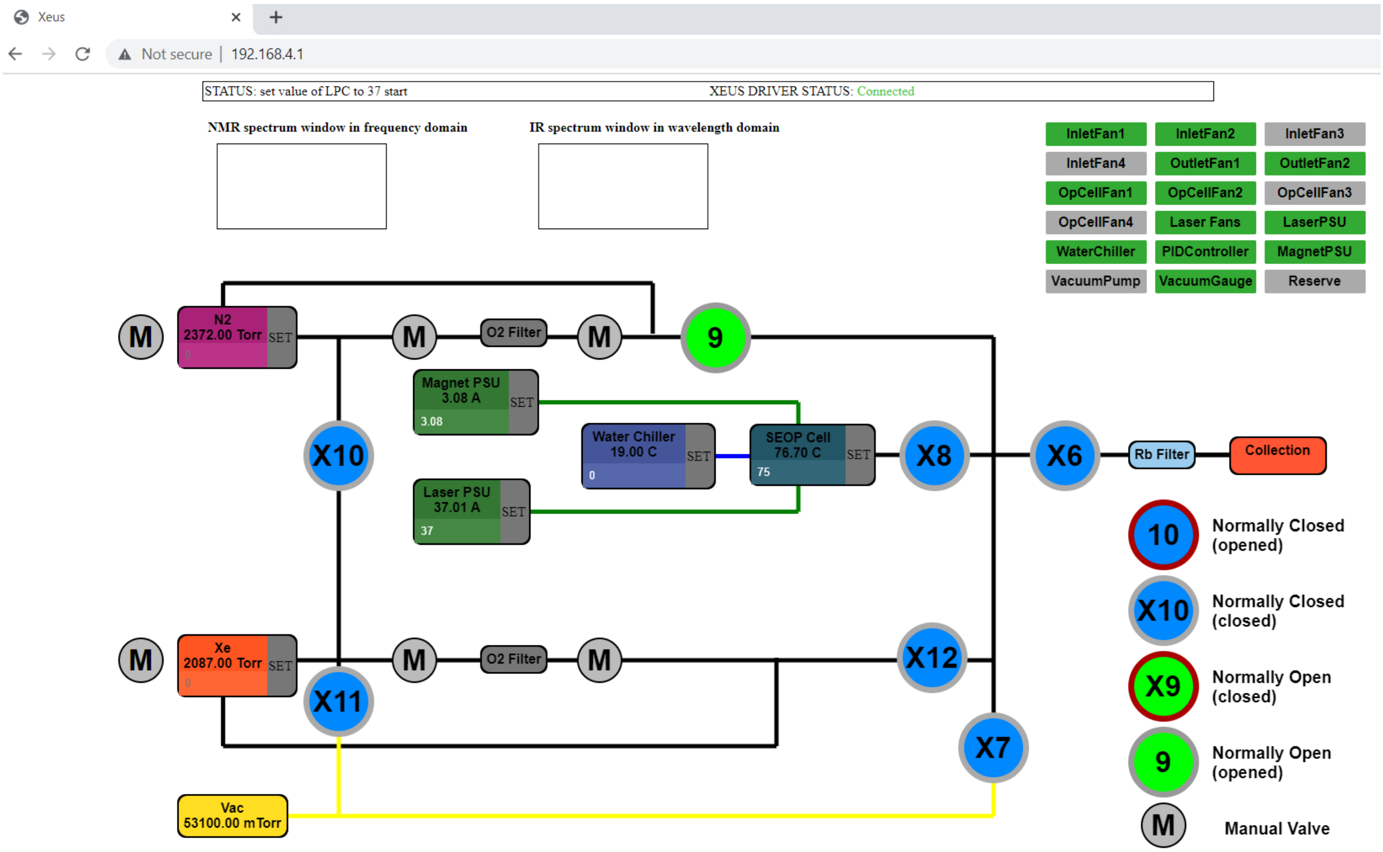

Figure S8: A representative state of the XeUS GEN-3 hyperpolarizer Graphical User Interface (GUI) while performing SEOP. Note valve #9 is normally open in the de-energized state.

9) Effect of RF Pulse Amplitude on "Apparent" P_{Xe}

As described in the main text, the excitation RF pulse strength B_1 for ^{1}H and ^{129}Xe nuclei is set to be the same by adjusting the voltages of the pulses to compensate for the ($\gamma_{1H}/\gamma_{129Xe}$) ratio (*i.e.*, necessitating ~3.6 times greater voltage for ^{129}Xe versus ^{1}H). In case if a greater ratio is employed (by mistake), the result can lead to substantial systematic error in the calculation of P_{Xe}. Figure S9 shows the build-up plot, where the initial data points were acquired using ~1.26-times stronger B_1 than the required ~3.6-fold ratio. After the steady state is achieved, the B_1 was corrected (data points labeled "-25.3 db") resulting in a sharp drop in the apparent P_{Xe}. Overall, the P_{Xe} was overestimated by a factor of 1.22 in this representative example, when a higher-than-required B_1 strength was employed to detect the HP ^{129}Xe signal.

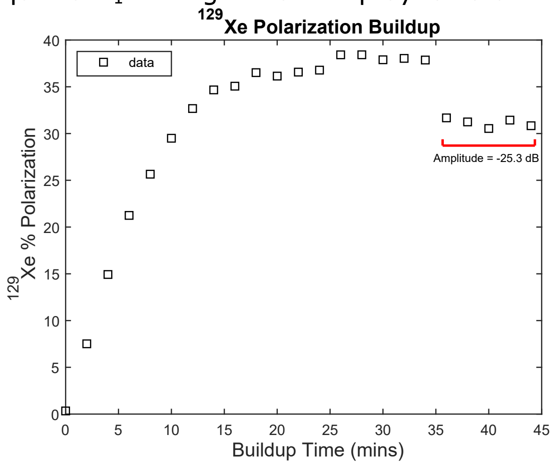

Figure S9: ^{129}Xe polarization buildup monitored by RF pulse duration of 150 μs with amplified gain of -23.3 db during the build-up process. Once the steady-state is achieved the amplitude of RF excitation pulse was corrected to -25.3 db (correct) value. Note the overestimation of P_{Xe} by a factor of 1.22-fold due to stronger B_1 strength.

10) Aluminum Jacket Extenders for the SEOP Cell

SEOP Cell **FRONT** SEOP Cell **BACK**

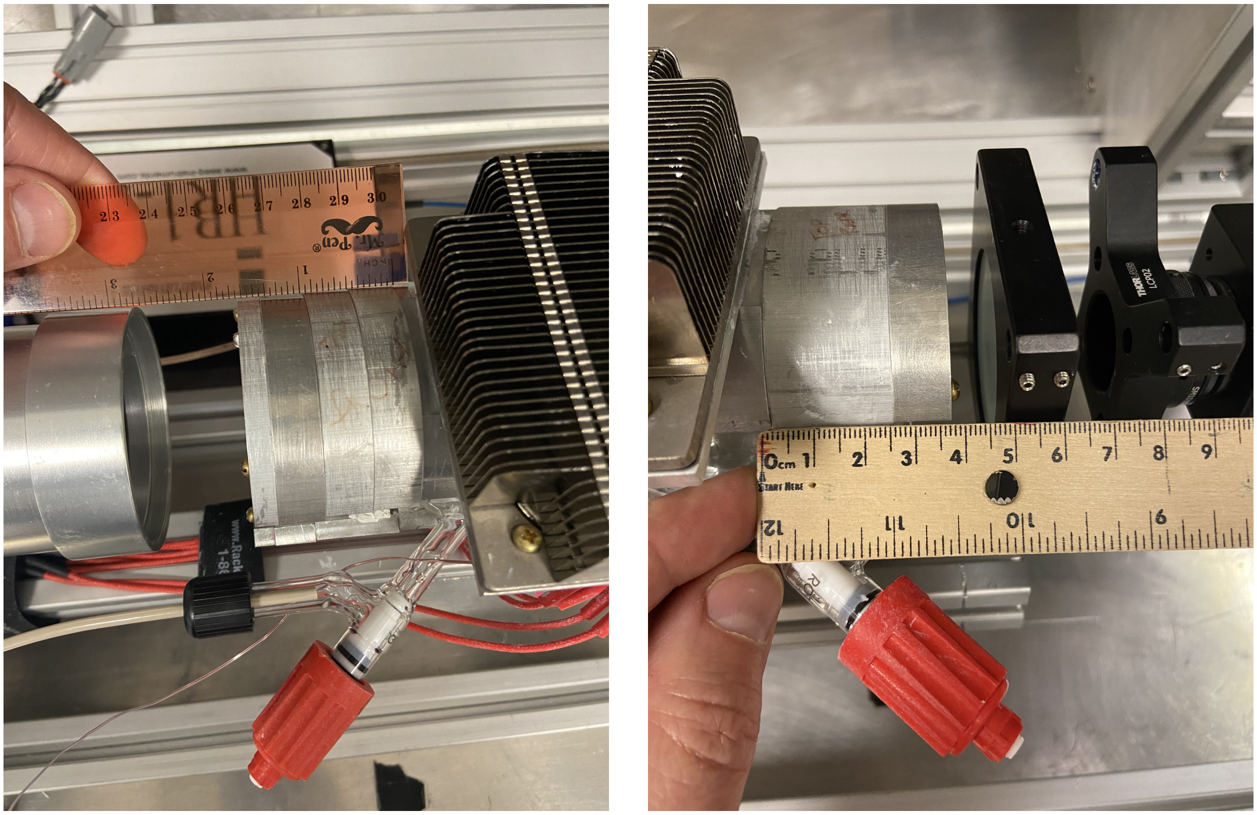

Figure S10: Photographs of the aluminum jacket extenders employed for the SEOP cell.

11) Schematic of Rb Distribution (a.k.a. “Spreader”) Setup

Please see Section 5 of SI for details.

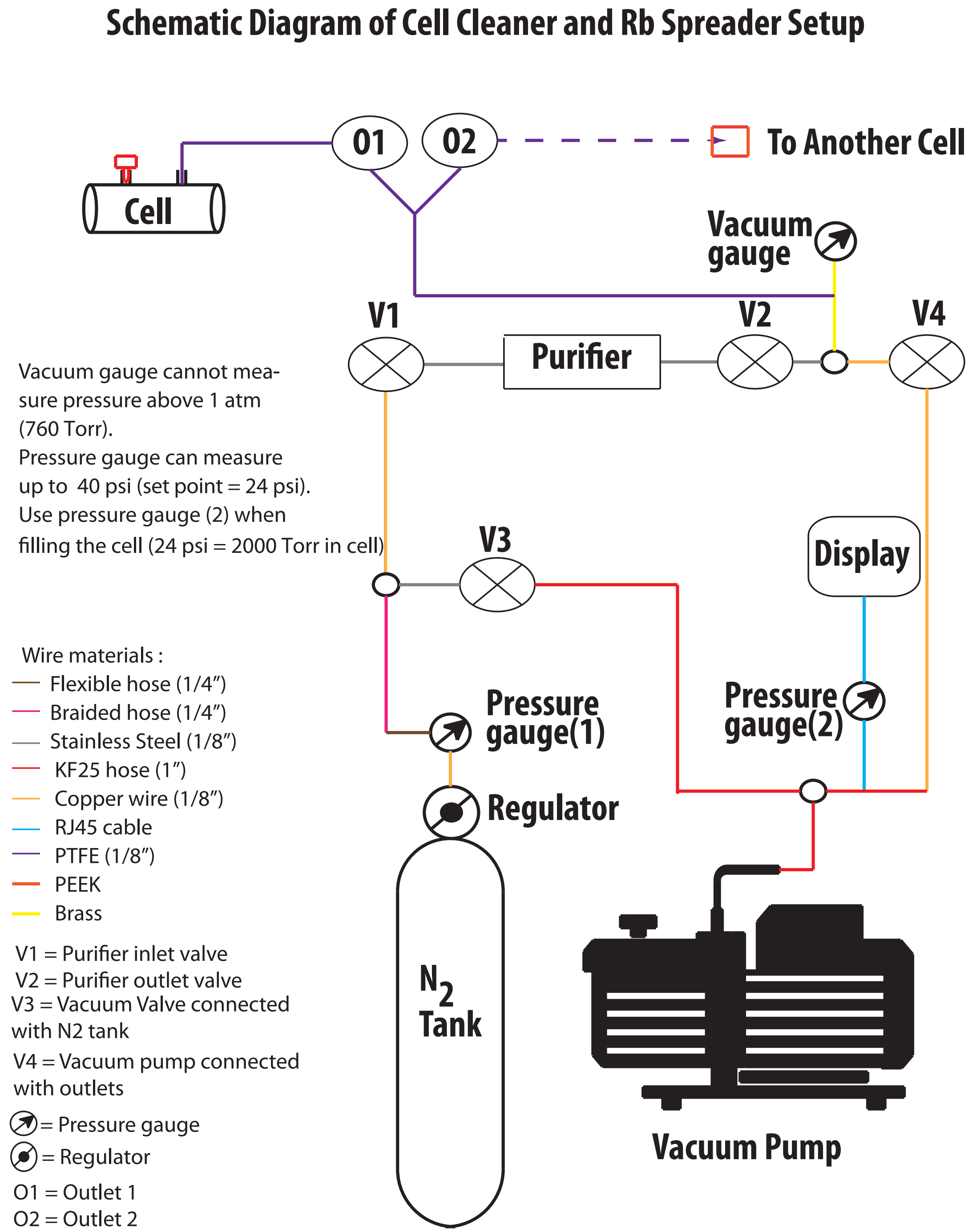

Figure S11: Schematic Diagram of SEOP Cell “Cleaner” and Rb distribution setup (a.k.a. the Rb “spreader”).

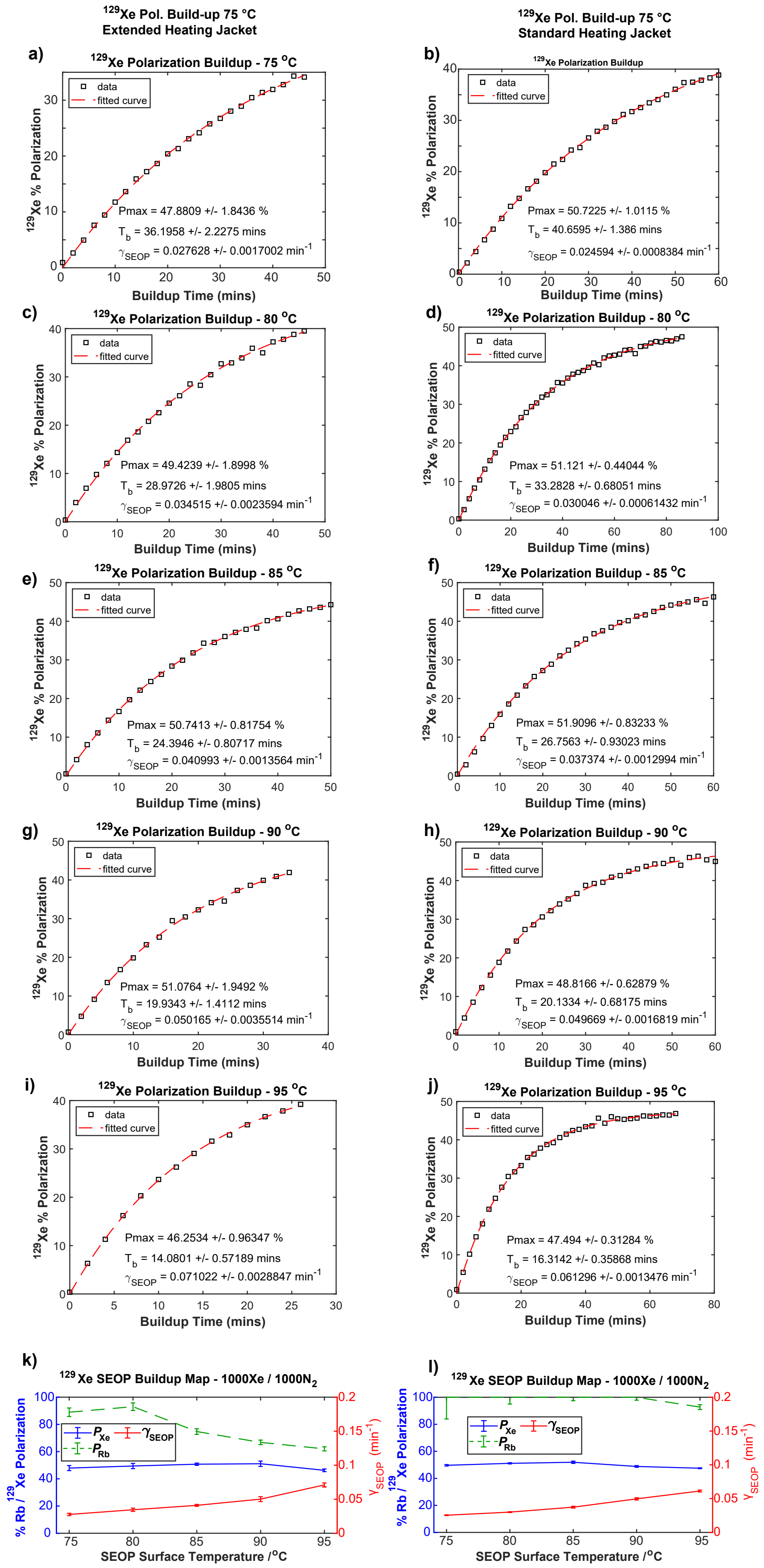

Figure S12: Comparison of ^{129}Xe polarization build-up at different temperatures and temperature map with extended jacket (a,c,e,g,i,k) and without the extended jacket (b,d,f,h,j,l).

12) References Used in Supporting Information

[1] J.R. Birchall, R.K. Irwin, M.R.H. Chowdhury, P. Nikolaou, B.M. Goodson, M.J. Barlow, A. Shcherbakov, E.Y. Chekmenev, Automated Low-Cost In Situ IR and NMR Spectroscopy Characterization of Clinical-Scale ^{129}Xe Spin-Exchange Optical Pumping, Anal. Chem., 93 (2021) 3883-3888.
[2] J.R. Birchall, P. Nikolaou, A.M. Coffey, B.E. Kidd, M. Murphy, M. Molway, L.B. Bales, B.M. Goodson, R.K. Irwin, M.J. Barlow, E.Y. Chekmenev Batch-Mode Clinical-Scale Optical Hyperpolarization of Xenon-129 Using an Aluminum Jacket with Rapid Temperature Ramping, Anal. Chem., 92 (2019) 4309-4316.
[3] J.R. Birchall, R.K. Irwin, P. Nikolaou, E. Pokochueva, K.V. Kovtunov, K.I. V., M.J. Barlow, B.M. Goodson, E.Y. Chekmenev Pilot Multi-Site Quality Assurance Study of Batch-Mode Clinical-Scale Automated Xenon-129 Hyperpolarizers, J. Magn. Reson., 316 (2020) 106755.
